# Fast tumor phylogeny regression via tree-structured dual dynamic programming

**DOI:** 10.1093/bioinformatics/btaf235

**Published:** 2025-07-15

**Authors:** Henri Schmidt, Yuanyuan Qi, Benjamin J Raphael, Mohammed El-Kebir

**Affiliations:** Department of Computer Science, Princeton University, Princeton, NJ 08542, United States; Siebel School of Computing and Data Science, University of Illinois Urbana-Champaign, Urbana, IL 61801, United States; Department of Computer Science, Princeton University, Princeton, NJ 08542, United States; Siebel School of Computing and Data Science, University of Illinois Urbana-Champaign, Urbana, IL 61801, United States

## Abstract

**Motivation:**

Reconstructing the evolutionary history of tumors from bulk DNA sequencing of multiple tissue samples remains a challenging computational problem, requiring simultaneous deconvolution of the tumor tissue and inference of its evolutionary history. Recently, phylogenetic reconstruction methods have made significant progress by breaking the reconstruction problem into two parts: a regression problem over a fixed topology and a search over tree space. While effective techniques have been developed for the latter search problem, the regression problem remains a bottleneck in both method design and implementation due to the lack of fast, specialized algorithms.

**Results:**

Here, we introduce *fastppm*, a fast tool to solve the perfect phylogeny regression problem via *tree-structured dual dynamic programming*. *fastppm* supports arbitrary, separable convex loss functions including the $\ell_{2}$, piecewise linear, binomial and beta-binomial loss and provides asymptotic improvements for the $\ell_{2}$ and piecewise linear loss over existing algorithms. We find that *fastppm* empirically outperforms both specialized and general purpose regression algorithms, obtaining 50–450× speedups while providing as accurate solutions as existing approaches. Incorporating *fastppm* into several phylogeny inference algorithms immediately yields up to 400× speedups, requiring only a small change to the program code of existing software. Finally, *fastppm* enables analysis of low-coverage bulk DNA sequencing data on both simulated data and in a patient-derived mouse model of colorectal cancer, outperforming state-of-the-art phylogeny inference algorithms in terms of both accuracy and runtime.

**Availability and implementation:**

*fastppm* is implemented in C++ and available as both a command-line interface and Python library at github.com/elkebir-group/fastppm.git under an MIT license.

## 1 Introduction

Inferring the evolutionary history of tumors, known as *phylogenetic reconstruction*, is a fundamental challenge in cancer genomics. Over the past two decades, phylogenetic reconstruction of tumors has been performed using a variety of sequencing technologies, including bulk DNA sequencing (Jamal-Hanjani *et al.* 2017), single-cell DNA sequencing (Laks *et al.* 2019, Minussi *et al.* 2021, Funnell *et al.* 2022), single-cell RNA sequencing (Zhou *et al.* 2020), and lineage tracing (Quinn *et al.* 2021, Yang *et al.* 2022) technologies. Concurrently, reconstruction has been performed using a variety of phylogenetic markers, such as somatic single-nucleotide variants (SNVs) (Malikic *et al.* 2015, El-Kebir *et al.* 2016, El-Kebir 2018, Kozlov *et al.* 2022, Wintersinger *et al.* 2022, Weber *et al.* 2023, Kulman *et al.* 2024), copy number aberrations (Kaufmann *et al.* 2022, Schmidt *et al.* 2023), gene expression (Zhou *et al.* 2020), and DNA methylation (Liu *et al.* 2023). Despite the diversity of sequencing technologies and markers used to perform phylogenetic reconstruction of tumors, reconstructing tumor phylogenies from bulk DNA sequencing of tumor tissue remains important, as large-scale cohort studies of patient tumors (Jamal-Hanjani *et al.* 2017, Gerstung *et al.* 2020, Fernandez-Mateos *et al.* 2024) continue to apply the technology.

A key difficulty in phylogenetic reconstruction of tumors from bulk DNA sequencing data is that sequencing measures a *mixture* of the underlying clonal genotypes. Effective methods for phylogenetic reconstruction (Malikic *et al.* 2015, El-Kebir *et al.* 2016, Satas and Raphael 2017, Myers *et al.* 2019, Wintersinger *et al.* 2022, Kulman *et al.* 2024, Schmidt and Raphael 2024) thus must simultaneously deconvolve the mixtures while performing tree reconstruction. However, it has recently been observed (Qi *et al.* 2019) that this phylogenetic reconstruction problem is highly under-determined, leading to non-uniqueness of solutions, i.e. the presence of multiple tumor phylogenies that explain the data equally well. Further, many tumor phylogeny methods approximate the raw read counts with estimated frequencies in order to speed up phylogenetic inference, leading to a loss of signal and accuracy. Finally, to manage computational intractability, many existing analyses and methods perform reconstruction on *clusters* of mutations (Gillis and Roth 2020), but this fails to leverage useful phylogenetic signal and relies on a correct clustering.

**Figure 1.**
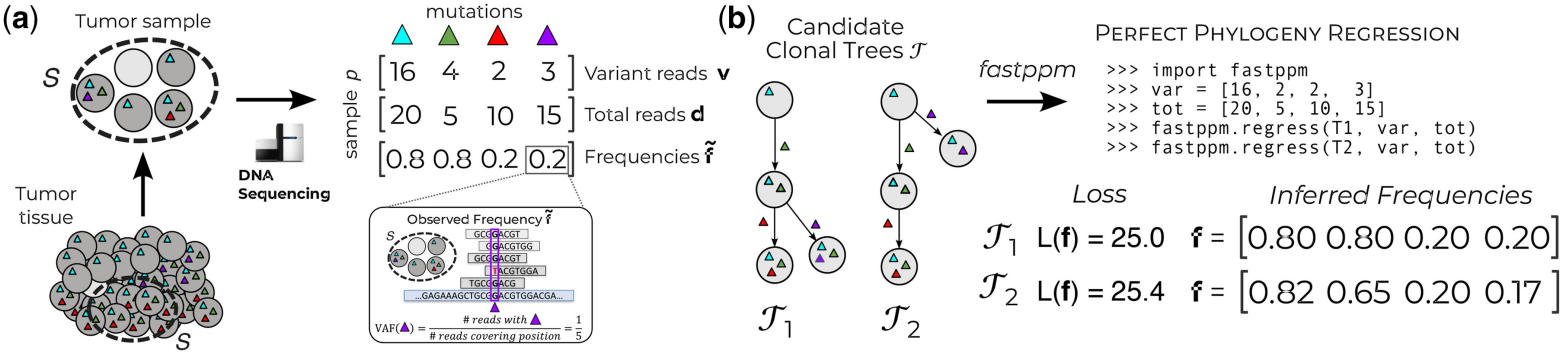
(a) Variant read counts v and total read counts d are obtained through bulk DNA sequencing of tumor tissue followed by sequence alignment and variant calling. (b) Provided a clonal tree T, variant read counts v, and total read counts d, *fastppm* solves the Perfect Phylogeny Regression problem to obtain the minimum loss along with inferred frequencies f, enabling the ranking of candidate clonal trees.

Envisioning that the next-generation of methods for phylogenetic reconstruction from bulk DNA sequencing will account for non-uniqueness of the solution space, accurately model read counts, and attempt reconstruction directly from mutation-level counts, we sought to build a framework promoting these developments. The key computational bottleneck hindering such development is the lack of specialized algorithms for a *regression* problem over a fixed topology. Specifically, all scalable methods (Wintersinger *et al.* 2022, Kulman *et al.* 2024, Schmidt and Raphael 2024) for phylogenetic reconstruction work by breaking the reconstruction problem into two parts, a repeatedly solved Perfect Phylogeny Regression (PPR) problem over a fixed topology and a search over tree space. In the PPR problem, one seeks latent mutation frequencies f for a fixed tree topology T best explaining the observed read counts v,d, quantified via a specified loss function L(f) (Fig. 1a and b). Current methods to solve the PRR problem either use general-purpose convex optimization packages or specialized solvers (Table 1). However, general-purpose convex optimization packages do not leverage the unique tree structure inherent to the PPR problem. On the other hand, solvers that leverage the tree structure have only been developed for restrictive loss functions L(f) operating directly on frequencies and ignoring additional information present in the read counts, such as the $\ell_{1}$ (Schmidt and Raphael 2024) and $\ell_{2}$ loss (Jia *et al.* 2018).

**Table 1.** Properties of algorithms for solving the Perfect Phylogeny Regression problem with a loss function of the form $L(f)=\sum_{i=1}^{n}L(f_{i})$ where *n* is the size of the clonal tree T, *d* is the maximum depth of T, $\bar{d}$ is the average depth of T, *r* is the maximum number of children in T, *k* is the parameter used in the *k*-PLA and PPLA(k,τ,σ) algorithms, and *K* is the number of iterations used in the alternating directions method of multipliers (ADMM) algorithm.^a^

Loss function $L(f_{i})$	Algorithmic technique	Complexity	References	Applications
$\ell_{1}$: $\left|f_{i}-{\tilde{f}}_{i}\right|$	IPM			AncesTree, SPRUCE, CALDER
	LP Duality	O(nd) [exp.: $O(n^{3/2}\;\text{log}\;n)$]	Schmidt and Raphael (2024)	fastBE
$\ell_{2}$: $w_{i}{\left(f_{i}-{\tilde{f}}_{i}\right)}^{2}$	IPM			CITUP
	Moreau’s Decomposition	$O(n^{2})$	Jia *et al.* (2018)	Pairtree, Orchard, EXACT
	TSDDP	$O(\min\{n^{2},n\bar{d}\;\text{log}\;r\})$	Here	CITUP*, Orchard*
		[exp.: $O(n^{3/2}\;\text{log}\;\;\text{log}\;n)$]		
Convex piecewise linear with *k* pieces	IPM			Sapling (Qi and El-Kebir 2024)
	TSDDP	$O(nk{\;\text{log}\;}^{2}(nk))$	Here	Sapling*
Binomial	IPM			Sapling (Qi and El-Kebir 2024)
	TSDDP-(P)PLA	$O(nk{\;\text{log}\;}^{2}(nk))$	Here	Sapling*
	TSDDP-ADDM	$O(K\min\{n^{2},n\bar{d}\;\text{log}\;r\})$	Here	Sapling*
Beta-binomial	TSDDP-(P)PLA	$O(nk{\;\text{log}\;}^{2}(nk))$	Here	
$L(f_{i})$ convex, β-Lipschitz	TSDDP-(P)PLA	$O(nk{\;\text{log}\;}^{2}(nk))$	Here	
	TSDDP-ADMM	$O(K\min\{n^{2},n\bar{d}\;\text{log}\;r\})$	Here	

a TSDDP refers to the tree structured dual dynamic programming technique introduced in this work. Moreau’s decomposition refers to the method for constructing the dual developed in (Jia *et al.* 2018, Ray *et al.* 2019). IPM refers to interior point methods which form the basis of state-of-the-art convex optimization software. The notation [exp.:] refers to the expected runtime over random classes of *n*-vertex trees (Addario-Berry and Donderwinkel 2024).

To overcome the key computational bottleneck in tumor phylogeny reconstruction from bulk DNA sequencing data, we developed *fastppm*. *fastppm* solves the Perfect Phylogeny Regression problem via tree-structured dual dynamic programming (TSDDP), a new technique that supports arbitrary loss functions of the form $L(f)=\sum_{i=1}^{n}L_{i}(f_{i})$ where $L_{i}$ is convex. This includes previously studied loss functions such as the $\ell_{1}$ and (weighted) $\ell_{2}$ loss, for which we obtain *asymptotic* improvements over existing algorithms (Jia *et al.* 2018, Schmidt and Raphael 2024), along with loss functions modeling commonly used probability distributions for read count data such as the binomial and beta-binomial distributions. We implemented *fastppm* in C++, and made it available as both a shared library and via a command-line interface. Moreover, *fastppm* provides Python bindings, making *fastppm* easy-to-use in existing tumor phylogeny inference pipelines (Fig. 1b). We demonstrate the advantages of *fastppm* on simulated tumors, showing speedups of 100× for the $\ell_{2}$ loss and 400× for the binomial loss. Incorporating *fastppm* into state-of-the-art tumor phylogeny methods such as CITUP (Malikic *et al.* 2015), Orchard (Kulman *et al.* 2024), and Sapling (Qi and El-Kebir 2024) consequently led to significant speed-ups of 3× to 400×. Specifically, *fastppm* enables Sapling to scale beyond 500 mutations, while the original implementation could only be run up to 50 mutations. On real data, we show that *fastppm* enables one to use accurate loss functions that operate on read counts rather than frequencies. This in turn enables more accurate tree inference, especially in a shallow coverage setting where one might opt to trade-off coverage for sequencing more regions/biopsies of a tumor.

## 2 Problem statement

We consider the problem of tumor phylogeny inference from bulk DNA sequencing data. After aligning sequencing reads to the reference genome and performing variant calling (Fig. 1a), we obtain the variant and total read count matrices $V,D\in N^{m\times n}$ of *n* single-nucleotide variants (SNVs) across *m* samples. In this manuscript, we will refer to SNVs as mutations. For each mutation *i* in sample *p*, we denote the number of variant and total reads by $v_{pi}$ and $d_{pi}$, respectively. In addition, the quantity ${\tilde{f}}_{pi}=v_{pi}/d_{pi}$ is the *observed frequency*, also known as the variant allele frequency of mutation *i* in sample *p*. We assume that the variant read counts $v_{pi}$ have been corrected for the effect of copy-number aberrations, so that ${\tilde{f}}_{pi}$ estimates the descendant cell fraction (Satas *et al.* 2021), or fraction of tumor cells in sample *p* that descend from the ancestral cell where mutation *i* was first gained.

The *n* mutations of the tumor evolve down a rooted tree, called a *clonal tree* T (El-Kebir *et al.* 2015, Schmidt and Raphael 2024), such that each mutation is gained exactly once and never subsequently lost—this is known as the infinite sites assumption (ISA) (Kimura 1969). We note that, while the infinite sites assumption may be violated for individual mutations due to copy-number loss, tumor phylogeny inference pipelines correct for such events by inferring mutation clusters for which the ISA holds (Jamal-Hanjani *et al.* 2017, Gillis and Roth 2020). We will consider mutation clusters as individual mutations. A mutation cluster C⊆[n] is summarized as an individual mutation *i* in one of two ways. First, one can pool reads, i.e. $v_{pi}:=\sum_{j\in C}v_{pj}$ and $d_{pi}:=\sum_{j\in C}d_{pj}$. Alternatively, to maintain a similar level of variance as the average mutation in the cluster, one can compute the average cluster VAF ${\tilde{f}}_{pi}=|C{|}^{-1}\sum_{j\in C}{\tilde{f}}_{pj}$ and set $d_{pi}:=|C{|}^{-1}\sum_{j\in C}d_{pj}$ followed by $v_{pi}:={\tilde{f}}_{pi}\cdot d_{pi}$, as done in Jamal-Hanjani *et al.* (2017). We assume that each ${\tilde{f}}_{pj}$ has been corrected for the effect of copy-number aberrations.

The *n* vertices or *clones* of T are uniquely identified by the mutation on the edge into the clone, and are labeled as [n]={1,…,n}. Associated with each clone *i* of T is a *n*-dimensional binary vector $b_{i}\in {\left\{0,1\right\}}^{n}$ recording the mutations present in clone *i*. Specifically, $b_{ij}=1$ if and only if mutation *j* is present in clone *i*. Given a clonal tree T, we denote its root node as r=r(T), its vertex set as V(T), and its edge set as E(T). Further, the parent (if it exists) of vertex *i* in T is denoted as π(i), its children as δ(i), and its descendants (including itself) as D(i).

As bulk DNA sequencing measures a mixture of clones, we denote with $u_{pi}\geq 0$ the proportion of clone *i* in sample *p*. Consequently, the underlying frequency $f_{pi}$ with which we observe mutation *i* in sample *p* is $f_{pi}=\sum_{j}u_{pj}b_{ji}$. Compactly, this relation can be written in matrix form as F=UB where $B=[b_{ij}]$ is the *n*-by-*n clonal matrix* associated with T, $F=[f_{pi}]$ is the *m*-by-*n frequency matrix*, and $U=[u_{pi}]$ is the *m*-by-*n usage matrix*. There is a one-to-one correspondence between clonal matrices B and clonal trees T (El-Kebir *et al.* 2015, Schmidt and Raphael 2024). Note that that the underlying frequency $f_{pi}$ might not be equal to the observed frequency ${\tilde{f}}_{pi}=v_{pi}/d_{pi}$ obtained from the observed variant and total read counts due to sampling uncertainty.

The goal in tumor phylogeny inference is to infer the true clonal tree T, the underlying frequencies F, and the usage matrix U, provided the read count data $V,D\in N^{m\times n}$. To infer and/or summarize tumor phylogeny solution spaces, existing methods repeatedly solve the following optimization problem for either different clonal trees T (Malikic *et al.* 2015, Ray *et al.* 2019, Schmidt and Raphael 2024) or in a progressive fashion by growing clonal trees one mutation at a time (El-Kebir *et al.* 2016, Myers *et al.* 2019, Wintersinger *et al.* 2022, Kulman *et al.* 2024, Qi and El-Kebir 2024). Moreover, existing methods use different loss functions (summarized in Table 1).

**Problem 1** (Perfect Phylogeny Regression (PPR)). *Given variant and total read counts* $V,D\in N^{m\times n}$  *for m samples and n mutations, a clonal matrix* $B\in {\left\{0,1\right\}}^{n\times n}$  *associated with a clonal tree* T  *and loss function*  $L(F|V,D):{\left[0,1\right]}^{m\times n}\to R_{\geq 0}$*, find a frequency matrix* $F\in {\left[0,1\right]}^{m\times n}$  *and usage matrix* $U\in {\left[0,1\right]}^{m\times n}$, U1≤1  *such that* F=UB  *and* L(F | V,D)  *is minimized.*

We denote the value of the minimizer as $L^{*}(T)$. When the loss function separates across samples as $L(F\;|\;V,D)=\sum_{p=1}^{m}L^{\prime }(f_{p}\;|\;v_{p},d_{p})$, which is typical, one can solve the PPR problem independently for each sample. Thus, we assume m=1 and use row vectors $f^{T}$ and $u^{T}$ rather than the matrices F and U.

## 3 Theory and algorithms

Although specific cases of the PPR problem have been studied in the tumor phylogenetics literature, no general theory has developed for solving phylogeny constrained regression problems. By using Lagrangian duality as a starting point, we start to develop such a theory, showing that the PPR problem is equivalent to a *tree separable* optimization problem (see Page 1). Exploiting this tree separability, we derive an abstract dynamic programming algorithm for solving the PPR problem, an approach we call *tree structured dual dynamic programming (TSDDP)*. A summary of the algorithmic results obtained with TSDDP is provided by Table 1.

### 3.1 A tree separable dual problem

We generalize the dual construction used in Schmidt and Raphael (2024) to derive an equivalent form of the PPR problem, which is *tree separable* in the coordinates of the dual variables, a term we define below. Rather than use linear programming (LP) duality, however, we use the much stronger notion of Lagrangian duality (Boyd and Vandenberghe 2004). Not only does this simplify the dual construction by allowing us to avoid an epigraphical reformulation of our problem, but it also allows us to extend the dual to arbitrary convex loss functions beyond the $\ell_{1}$ norm. As we will demonstrate in the next section, the tree separability of the dual problem (see Page 1) allows for efficient optimization using a tree structured dynamic programming algorithm.

To start, fix a clonal matrix B and its associated clonal tree T, and assume the loss function *L* separates across mutations as $L(f\;|\;v,d)=\sum_{i=1}^{n}L_{i}(f_{i}\;|\;v_{i},d_{i})$. Note that this assumption typically follows directly from independence among the *n* mutations. Simplifying notation by omitting the variant and total read counts from the loss function, the PPR problem can be written mathematically as the following (primal) optimization problem,


(1) $$\underset{f,u\in R^{n}}{\min}\{\sum_{i=1}^{n}L_{i}(f_{i}):f^{T}=u^{T}B,u\geq 0,u^{T}1\leq 1\}.$$

To simplify the preceding optimization problem, we invoke the basic theory of clonal matrices and trees developed in El-Kebir *et al.* (2015) and Schmidt and Raphael (2024), which states that for a given frequency vector $f^{T}$ there exists a usage vector $u^{T}$ such that $f^{T}=u^{T}B$ if and only if f satisfies the following Sum Condition (SC),


(SC) $$f_{i}\geq \sum_{j\in \delta (i)}f_{j}\;\text{for}\;\text{all}\;i\in [n].$$

More formally, we use the following result, which can be proven both combinatorially (El-Kebir *et al.* 2015) or algebraically (Schmidt and Raphael 2024).

Proposition 1. *Let* T  *be a clonal tree and* B  *be its associated clonal matrix. Then, for a frequency vector* $f^{T}$  *there exists a usage vector* $u^{T}$  *such that* u≥0, $u^{T}1\leq 1$*, and* $f^{T}=u^{T}B$  *if and only if* f  *satisfies the Sum Condition (SC) and has* $f_{r}\leq 1$.

Applying Proposition 1 to the primal problem (1), we obtain the equivalent optimization problem which factors out the usage vector u:


(2) $$p^{*}:=\underset{f\in R^{n}}{\min}\{\sum_{i=1}^{n}L_{i}(f_{i}):f_{r}\leq 1,f\;\text{satisfies}\;(\text{SC})\}.$$

As B is invertible [in linear time (Schmidt and Raphael 2024)], we can recover the unique $u^{*}$ corresponding to the minimizer $f^{*}$ in O(n) time.

To derive the Lagrangian dual, we associate the dual vector $\alpha =(\alpha_{0},\alpha_{1},\ldots ,\alpha_{n})\in R_{\geq 0}^{n+1}$ with the inequality constraints, obtaining the Lagrangian,


(3) $$\begin{array}{c} L(f,\alpha ):=\sum_{i=1}^{n}L_{i}(f_{i})-\sum_{i=1}^{n}[\alpha_{i}(f_{i}-\sum_{j\in \delta (i)}f_{j})]-\alpha_{0}(1-f_{r})\\ =-\alpha_{0}+\sum_{i=1}^{n}\left[L_{i}(f_{i})-(\alpha_{i}-\alpha_{\pi (i)})f_{i}\right]. \end{array}$$

Here, the equality follows from the fact that each $f_{i}$ appears twice in the right hand side of (3): once with $\alpha_{i}$ and once with the parent dual variable $\alpha_{\pi (i)}$. For simplicity, we are abusing notation and setting π(r)=0.

The Lagrangian dual function g(α), then optimizes out the primal variable f, yielding $g(\alpha ):=-\alpha_{0}+\sum_{i=1}^{n}h_{i}(\alpha_{i}-\alpha_{\pi (i)})$ where $h_{i}(x):=\min_{f}\{L_{i}(f)-xf\}$. Importantly, the dual function g(α) is *tree separable* in the dual variable α. We will see in the next section that the tree separability of g(α) enables us to solve the Lagrangian dual problem with tree structured dynamic programming. The Lagrangian dual problem is $d^{*}:=\max_{\alpha \geq 0}g(\alpha )$. Since α≥0, it is clear that $d^{*}\leq p^{*}$ for an arbitrary (not necessarily convex) set of loss functions $L_{i}$. However, when $L_{i}$ are convex, we obtain strong duality $d^{*}=p^{*}$ as the constraint set is affine (Boyd and Vandenberghe 2004). To complete this section, we summarize our results in the following theorem statement.

Theorem 1. *Let* T  *be a clonal tree on n mutations. If the loss function separates as* $L(f)=\sum_{i=1}^{n}L_{i}(f_{i})$  *with* $L_{i}$  *all convex, then the PPR problem is equivalent to* $\max_{\alpha \in R^{n+1}}\{-\alpha_{0}+\sum_{i=1}^{n}h_{i}(\alpha_{i}-\alpha_{\pi (i)}):\alpha \geq 0\}$*, where* $h_{i}(x):=\min_{f}\{L_{i}(f)-xf\}$.

### 3.2 Tree structured dual dynamic programming

We provide a meta algorithm for solving the tree separable dual problem using *tree structured dual dynamic programming (TSDDP)*. We describe the algorithm’s basic ingredients and several properties that are conserved across different choices of loss function.

To start, let $J_{i}$ be the optimal solution to the PPR problem for the subtree rooted at node *i* when the dual variable of the parent $\alpha_{\pi (i)}$ is fixed. Formally, let 


(4) $$J_{i}(\gamma ):=\underset{\alpha \geq 0}{\max}\{\sum_{j\in D(i)}h_{j}(\alpha_{j}-\alpha_{\pi (j)}):\alpha_{\pi (i)}=\gamma \}.$$

To solve the PPR problem it is sufficient to maximize over $J_{r}$ since $d^{*}=\max_{\alpha_{0}\geq 0}\{-\alpha_{0}+J_{r}(\alpha_{0})\}$ by definition. Due to the tree separability of the PPR problem, however, $J_{r}$ is computable from $J_{i}$ over the children i∈δ(r). Indeed, this holds recursively, yielding the following recursion:


(5) $$J_{i}(\gamma )=\underset{\alpha_{i}\geq 0}{\max}\{h_{i}(\alpha_{i}-\gamma )+\sum_{j\in \delta (i)}J_{j}(\alpha_{i})\}.$$

The above recurrence then implies the following bottom-up dynamic programming algorithm for computing the optimal value of PPR problem.

- Fix a representation $R(J_{i})$ for each $J_{i}$.
- Compute the representation $R(J_{i})$ at the leaf nodes.
- Compute the representation $R(J_{i})$ at a node *i* provided the representations $R(J_{j})$ at all children j∈δ(i).
- Solve the 1D optimization problem $\max_{\alpha_{0}\geq 0}\{-\alpha_{0}+J_{r}(\alpha_{0})\}$ using the representation of the root node $R(J_{r})$.

To recover the optimal solution, we perform top-down backtracking over the tree topology, analogous to the backtracking performed to recover an optimal ancestral labeling in Sankoff’s algorithm (Sankoff and Rousseau 1975). In particular, we recover the dual variable $\alpha_{0}^{*}=\text{arg}{\text{max}}_{\alpha_{0}\geq 0}\{-\alpha_{0}+J_{r}(\alpha_{0})\}$. Then, given the optimal dual variable $\alpha_{\pi (i)}^{*}$ of the parent π(i) of *i*, we compute the optimal dual variable for node *i* as


(6) $$\alpha_{i}^{*}=\underset{\alpha_{i}\geq 0}{\text{arg max}}\{h_{i}(\alpha_{i}-\alpha_{\pi (i)}^{*})+\sum_{j\in \delta (i)}J_{j}(\alpha_{i})\},$$

where we set $\alpha_{\pi (r)}=\alpha_{0}$ for convenience. Since the representations of $J_{j}$ have already been computed prior to backtracking, recovering the dual variables consists of solving *n* 1D optimization problems. We finally recover the primal variables $f_{i}^{*}$ by computing $f_{i}^{*}=\text{arg}{\text{min}}_{f_{i}}\{L_{i}(f_{i})-(\alpha_{i}^{*}-\alpha_{\pi (i)}^{*})\cdot f_{i}\}.$

In general, it is step (iii) of the TSDDP algorithm, the inductive step, which is the most challenging to implement, since one requires that the representation $R(J_{i})$ is closed under the recurrence relation. However, even in the general case, $J_{i}$ possess several nice properties. In particular, $h_{i}$ is the negation of the convex conjugate of $L_{i}$, implying that $h_{i}$ is concave, even when $L_{i}$ is not necessarily convex (Observation 1). Next, it holds that $J_{i}$ is always concave (Observation 2), implying that computing the 1D maximizer of $J_{i}$ is tractable.

### 3.3 Specializing TSDDP to $\ell_{1}$, $\ell_{2}$, and piecewise linear loss functions

Perhaps the simplest loss function is a quadratic, or $\ell_{2}$, loss. Indeed, one of the earliest methods for inferring tumor phylogenies from bulk DNA sequencing data, CITUP (Malikic *et al.* 2015), used a quadratic loss function within a quadratic integer programming framework. The quadratic loss generalizes to probabilistic settings where the observed frequencies are drawn from a Gaussian distribution centered at the estimated frequencies (Wintersinger *et al.* 2022, Kulman *et al.* 2024). Jia *et al.* (2018) solved the PPR problem under an unweighted $\ell_{2}$ loss with an $O(n^{2})$ time algorithm by constructing a dual problem using Moreau’s decomposition and solving it iteratively.

With TSDDP, we derive an asymptotically faster algorithm for the weighted $\ell_{2}$ loss. To derive this algorithm, we fix our computational representation $R(J_{i})$ to be the value $J_{i}(0)$, along with the intercept, slopes, and breakpoints of the piecewise linear derivative $J_{i}^{\prime }$. Computing the representation of the leaf nodes (ii) is easily done in O(1) time in closed form. The inductive step (iii) takes O( | D(i) |  log  | δ(i) | ) time and the final 1D optimization step (iv) takes O(n) time, leading to the desired time complexity using a careful analysis. The key technical challenge in obtaining our result is in proving that $J_{i}$ has a piecewise linear derivative and that the representation of the derivative $J_{i}^{\prime }$ is quickly computed. Detailed derivations for each step of the algorithm are provided in Results, available as supplementary data at *Bioinformatics* online.

Theorem 2. *TSDDP solves the PPR problem for the (weighted)* $\ell_{2}$  *loss* $L(f)=\sum_{i=1}^{n}w_{i}{\left(f_{i}-{\tilde{f}}_{i}\right)}^{2}$  *in* $O(\min\{n^{2},n\bar{d}\;\text{log}\;r\})$  *time where* $w\in R_{\geq 0}^{n}$  *are fixed weights*, $\bar{d}:=\frac{1}{n}\sum_{i=1}^{n}d_{i}$  *is the average depth of a node in* T*, and* $r:=\max_{i=1}^{n}\;|\;\delta (i)\;|\;$  *is the maximum out-degree.*

Thus, we improve upon the $O(n^{2})$ complexity of the Jia *et al.* (2018) algorithm. Another key advantage of our algorithm is that *almost all* trees have $O(\sqrt{n})$ diameter and O(log n) maximum out-degree under graph theoretic models of random trees (Addario-Berry and Donderwinkel 2024). In addition, when trees are restricted to being binary and are generated under a branching process model, they typically have O(log n) diameter (Devroye 1987). Consequently, our algorithm for the $\ell_{2}$ loss has $O(n^{3/2}\;\text{log}\;\;\text{log}\;n)$ expected complexity for random trees and O(n log n) expected complexity for random binary trees. The worst-case $O(n^{2})$ runtime appears when T is a path (i.e. $\bar{d}\approx \frac{n}{2}$).

Arguably, the next simplest loss function is the $\ell_{1}$ loss, which is found in several methods (El-Kebir *et al.* 2015, 2016, Myers *et al.* 2019, Schmidt and Raphael 2024) for inferring tumor phylogenies from bulk DNA sequencing data. To solve the PPR problem under an unweighted $\ell_{1}$ loss, our previous work (Schmidt and Raphael 2024) developed an algorithm running in O(nd) time, where *d* is the depth of T, by exploiting linear programming (LP) duality and the geometry of the $\ell_{1}$ loss.

Using TSDDP, we extend the case of an unweighted $\ell_{1}$ loss to convex piecewise linear loss functions, while also reducing the time complexity to $O(nk{\;\text{log}\;}^{2}(nk))$. To derive this algorithm, we fix (i) the computational representation $R(J_{i})$ to be the slopes, breakpoints, and intercept of $J_{i}$, which is shown to be a (concave) piecewise linear function. The key technical insight in obtaining the improved runtime is observing that the representations $R(J_{i})$ can be efficiently manipulated by storing the breakpoints and slopes in a specialized data structure which supports the addition and infimal conjugation of convex, piecewise linear functions (Tseng and Luo 1996). Using such a data structure, steps (ii) and (iii) take an average of $O(k{\;\text{log}\;}^{2}(nk))$ time. As the final 1D optimization step (iv) consists of evaluating the representation $J_{r}$ at all breakpoints, this leads the desired time complexity of $O(nk{\;\text{log}\;}^{2}(nk))$. Detailed derivations are provided in Results, available as supplementary data at *Bioinformatics* online.

Theorem 3. *TSDDP solves the PPR problem for a convex, piecewise linear loss function*  $L(f)=\sum_{i=1}^{n}\sum_{j=1}^{k}\left(c_{i,j}+m_{i,j}f_{i}\right)\cdot 1(f_{i}\in [x_{i,j-1},x_{i,j}))$  *in* $O(nk{\;\text{log}\;}^{2}(nk))$  *time where* $x_{i,j}$  *give the k breakpoints of* L(f)  *with respect to each* $f_{i}$.

### 3.4 Extending TSDDP to arbitrary loss functions

A key advantage of solving the PPR problem for the convex quadratic and piecewise linear loss functions is that it enables approximation of non-linear (convex) loss functions. Here, we present two such approaches to solving PRR for arbitrary convex loss functions using *fastppm* for the special cases of the $\ell_{2}$ and piecewise linear loss functions as a subroutine. We then apply these algorithms to the binomial and beta-binomial loss functions.

#### 3.4.1 A structural alternating directions method of multipliers approach

The alternating directions method of multipliers (ADMM) (Boyd 2010) reduces a constrained convex optimization to a sequence of *simpler* convex subproblems. It has recently been observed (Rontsis *et al.* 2022) that properly applying ADMM to difficult convex optimization problems results in fast algorithms able to exploit problem structure. Here, we apply ADMM to the PPR problem resulting in an algorithm which alternately solves (i) *n* 1D convex optimization problems and (ii) the $\ell_{2}$ case of PPR.

We start with the primal form (1) of PPR and replace the hard constraint u≥0, $u^{T}1\leq 1$ with the convex indicator function *g* where g(u)=0 if u≥0, $u^{T}1\leq 1$, and g(u)=∞ otherwise. Then, we obtain the following equivalent form of the PPR problem:


(7) $$\underset{f,u\in R^{n}}{\min}\{\sum_{i=1}^{n}L_{i}(f_{i})+g(u):B^{T}u=f\}.$$

Following (Boyd 2010), we introduce the *augmented Lagrangian*  $L_{\rho }(u,f,y):=\sum_{i=1}^{n}L_{i}(f_{i})+g(u)+y^{T}(B^{T}u-f)+(\rho /2)\;|\;\;|\;B^{T}u-f\;|\;{\;|\;}_{2}^{2},$ where ρ>0 is a scalar hyperparameter and $y\in R^{n}$ is the dual variable. By Lagrangian duality theory, solving (7) is equivalent to finding a saddle point of $L_{\rho }$. To find such a point, ADMM uses an iterative algorithm which at each iteration, minimizes f, then u, and finally updates the dual variable y in an ascent step. Specifically, ADMM performs the following iterations starting at an initial solution $\left(u^{\left(0\right)},f^{\left(0\right)},y^{\left(0\right)}\right)$:


$$\begin{array}{c} f^{\left(t+1\right)}=\underset{f}{\text{arg min}}\left\{\sum_{i=1}^{n}L_{i}(f_{i})+\frac{\rho }{2}\;|\;\;|\;B^{T}u^{\left(t\right)}-f+w^{\left(t\right)}\;|\;{\;|\;}_{2}^{2}\right\}\\ u^{\left(t+1\right)}=\underset{u}{\text{arg min}}\left\{g(u)+\frac{\rho }{2}\;|\;\;|\;B^{T}u-f^{\left(t+1\right)}+w^{\left(t\right)}\;|\;{\;|\;}_{2}^{2}\right\}\\ w^{\left(t+1\right)}=w^{\left(t\right)}+B^{T}u^{\left(t+1\right)}-f^{\left(t+1\right)}, \end{array}$$

where $w^{\left(t\right)}:=\frac{1}{\rho }y^{\left(t\right)}$ is the rescaled dual variable.

The f-update, by separability of the objective, consists in solving *n* 1D convex minimization problems. The u-update consists of solving the PPR problem for the $\ell_{2}$ loss $L_{i}(f_{i})={\left(f_{i}-{\tilde{f}}_{i}\right)}^{2}$ where ${\tilde{f}}_{i}=f_{i}^{\left(t\right)}-w_{i}^{\left(t\right)}$ is a perturbation of the frequency at the $t^{\text{th}}$ iteration. The w-update consists only of a matrix-vector product, which, by the combinatorial properties of B (Schmidt and Raphael 2024), takes O(n) time to perform.

#### 3.4.2 A piecewise linear approximation approach

Approximation of non-linear functions with piecewise linear loss functions is a well-studied technique in both the optimization (Geißler *et al.* 2011) and neural network literature (Hanin 2019), and is justified by a universal approximation theorem which states that arbitrary convex functions can be approximated by convex piecewise linear functions with a small number of pieces.

Given a set of differentiable, convex loss functions $L_{i}$, we provide two algorithms for solving the PPR problem using piecewise linear approximation. The first algorithm, called *Piecewise Linear Approximation with k segments* (*PLA-k*), performs the following steps:

1. Select k−1 breakpoints $x_{1}=\frac{1}{k},\ldots ,x_{k-1}=\frac{k-1}{k}$.
2. Get 1${}^{\text{st}}$-order Taylor expansion $g_{i,j}$ of $L_{i}$ at each $x_{j}$.
3. Define the piecewise linear approximation ${\tilde{L}}_{i}$ as the pointwise maximum over the Taylor approximations: ${\tilde{L}}_{i}(f_{i})=\max_{1\leq j\leq k}g_{i,j}(f_{i})$.
4. Solve the PPR problem using TSDDP with piecewise linear loss functions ${\tilde{L}}_{i}$.

From the definition of convexity, it follows that ${\tilde{L}}_{i}$ underestimates $L_{i}$ and converges to $L_{i}$ pointwise as k→∞. The total runtime of PLA-*k* is dominated by the final step, leading to a time complexity of $O(nk{\;\text{log}\;}^{2}(nk))$. Further, using a standard argument, we quantify the error of PLA-*k* for Lipschitz continuous loss functions $L_{i}$, as stated in the following theorem.

Theorem 4. *Let* T  *be a clonal tree on n mutations. Suppose* $L_{i}$  *is* β*-Lipschitz continuous and* ${\tilde{L}}_{i}$  *was constructed using PLA-k. Then*, $\;|\;L^{*}(T)-{\tilde{L}}^{*}(T)\;|\;\leq \frac{n\beta }{k}.$

The second algorithm, called *Progressive Piecewise Linear Approximation* (*PPLA*), is slightly more sophisticated, instead *progressively* performing piecewise linear approximation, updating the frequency bounds at each step of the algorithm (see Methods, available as supplementary data at *Bioinformatics* online).

## 4 Results

### 4.1 Fast and accurate regression using *fastppm*

We evaluated the performance of *fastppm* against five existing methods for the Perfect Phylogeny Regression problem under an $\ell_{2}$ and binomial loss. For both losses, we compared *fastppm* against the state-of-the-art convex programming solvers CVXOPT (Andersen *et al.* 2013), ECOS (Domahidi *et al.* 2013), Mosek (MOSEK ApS 2024), and Clarabel (Goulart and Chen 2024). For the $\ell_{2}$ loss, we additionally compared against the projectppm solver developed in (Jia *et al.* 2018, Ray *et al.* 2019). To evaluate each method, we simulated 90 tumor phylogenies, measuring the runtime and loss of the inferred frequencies (see Methods, available as supplementary data at *Bioinformatics* online for details). In addition to the observed frequencies (for $\ell_{2}$ loss) or the variant and total read counts (for binomial loss), all methods were provided the ground-truth simulated tree and were run in single-threaded mode on a 2.4 GHz CPU with 4 GB RAM.

**Figure 2.**
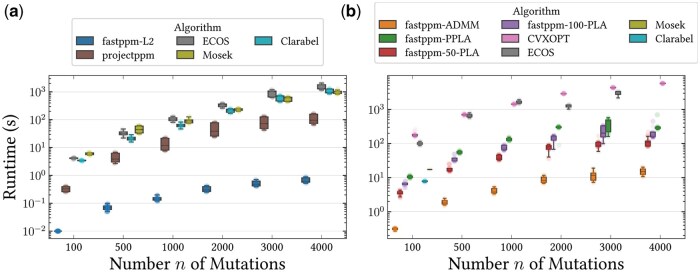
Wall-clock runtime of existing algorithms for the Perfect Phylogeny Regression problem across 90 tumor phylogenies for the (a) $\ell_{2}$ and (b) binomial loss functions. For the $\ell_{2}$ loss, all methods inferred the same optimal solution whereas Fig. 2, available as supplementary data at *Bioinformatics* online shows the objective value differences across methods for the binomial loss.

In terms of the $\ell_{2}$ loss, *fastppm* was on average 111.1× faster than projectppm (Fig. 2a), ranging from 33.3× faster when the number of mutations was small (n=100) to 163.2× faster when the number of mutations was large (n=4000). Compared to general-purpose convex programming solvers, *fastppm* yielded another order of magnitude improvement, outperforming the commercial solver Mosek by 858.49× on average (Fig. 2a). Since the $\ell_{2}$ loss is strictly convex and all solvers exactly solve the optimization problem, the inferred objective values were identical.

For the binomial loss, conic programming solvers ECOS (Domahidi *et al.* 2013), Mosek (MOSEK ApS 2024), and Clarabel (Goulart and Chen 2024) struggled to terminate without error on the vast majority of instances (54/270, see Fig. 1, available as supplementary data at *Bioinformatics* online). Although CVXOPT (Andersen *et al.* 2013) terminated on all instances, all three modes of *fastppm* were several orders of magnitude faster (Fig. 2b). Specifically, *fastppm*-ADDM, *fastppm*-50-PLA, *fastppm*-100-PLA, and *fastppm*-PPLA were a mean of 400.1×, 43.8×, 22.3×, and 13.6× faster than CVXOPT, respectively. The loss of the solutions inferred by *fastppm*-ADMM, *fastppm*-50-PLA, and *fastppm*-100-PLA was at most 4% higher than that inferred by CVXOPT, while the loss inferred by *fastppm*-PPLA was similar or slightly lower than that inferred by CVXOPT (Fig. 2, available as supplementary data at *Bioinformatics* online).

**Figure 3.**
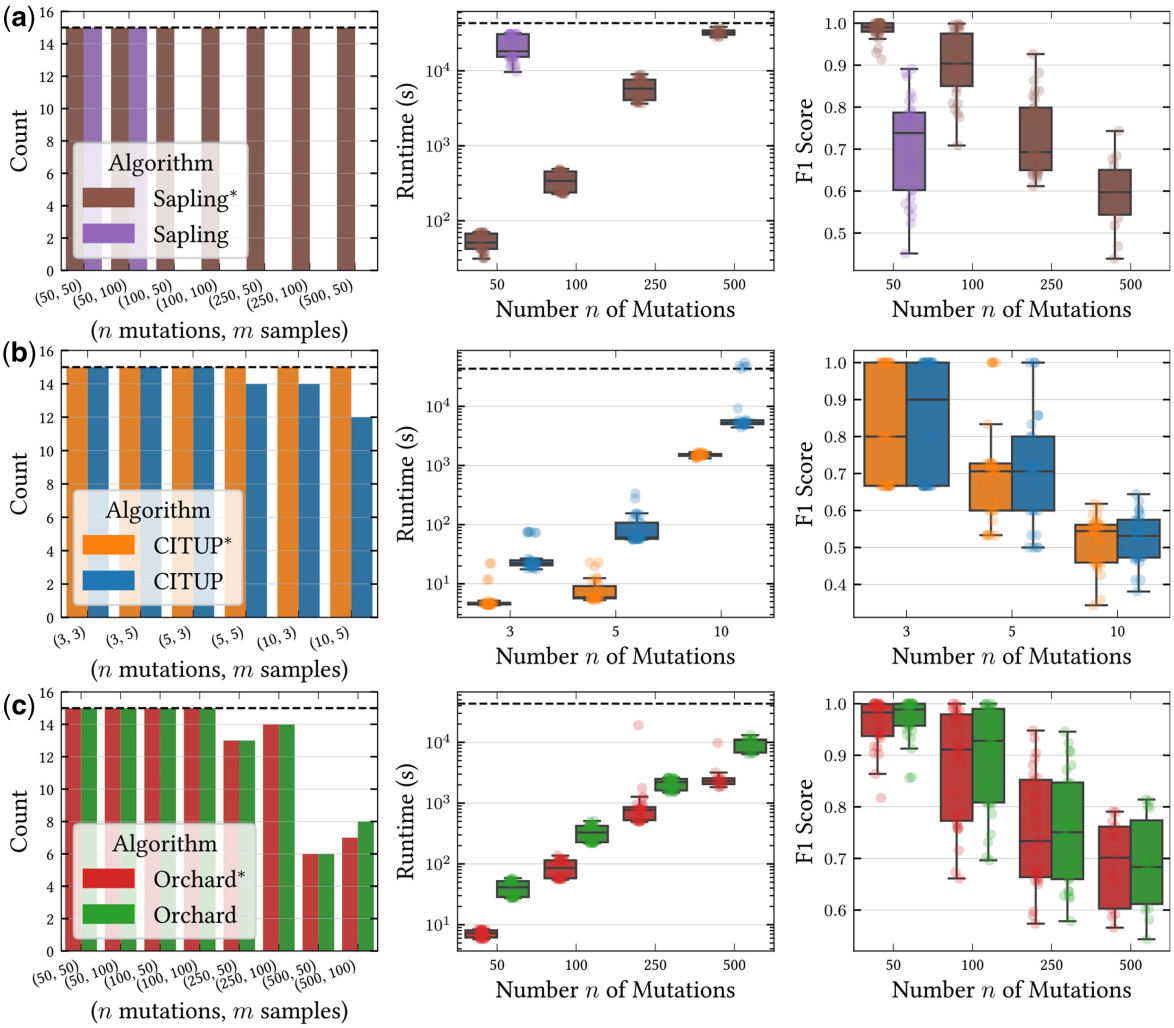
Number of successes within a 12-h time limit, wall-clock runtime (s), and tree inference accuracy across three different methods. (a) Sapling* is the result of replacing the CVXOPT solver for the binomial loss in Sapling (Qi and El-Kebir 2024) with *fastppm*-ADMM. (b) CITUP* is the result of replacing the CPLEX solver in CITUP (Malikic *et al.* 2015) for the $\ell_{2}$ loss with *fastppm*-L2. (c) Orchard* is the result of replacing the projectppm (Jia *et al.* 2018) solver in Orchard (Kulman *et al.* 2024) for the $\ell_{2}$ loss with *fastppm*-L2.

In summary, *fastppm* was at least an order of magnitude faster than existing methods while achieving nearly identical solutions on all problem instances (Fig. 2). In addition, we compared the binomial and beta-binomial loss implemented using fastppm-PPLA on overdispersed simulation data (i.e. with increased variance relative to a binomial distribution), finding that a beta-binomial loss better infers ground-truth frequencies (Fig. 3, available as supplementary data at *Bioinformatics* online).

### 4.2 Improved tree inference using *fastppm*

While the previous experiments assessed *fastppm* on a fixed ground-truth tree, we next evaluated *fastppm’*s ability to improve upon three existing phylogenetic inference pipelines: Sapling (Qi and El-Kebir 2024), CITUP (Malikic *et al.* 2015), and Orchard (Kulman *et al.* 2024). Specifically, we modified the existing software for all three tools by replacing calls to existing solvers with calls to *fastppm*, obtaining three new tools: Sapling*, CITUP*, and Orchard*. The modifications consisted of fewer than fifty lines of code in each case. For each altered method, we compared the runtimes and distances to the ground-truth tree using two distance measures, the parent-child distance and the ancestor-descendant distance (Govek *et al.* 2018) (details in Methods, available as supplementary data at *Bioinformatics* online).

**Figure 4.**
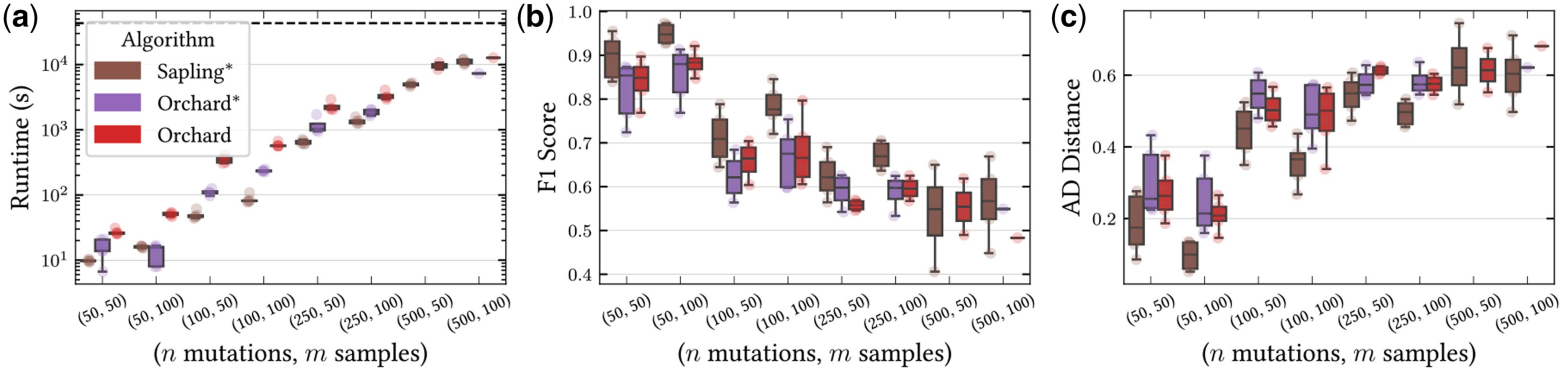
(a) Wall-clock runtime (s), (b) F1 score, and (c) ancestor-descendant distance for the phylogenies inferred by Sapling*, Orchard*, and Orchard (Kulman *et al.* 2024) on simulated 20× coverage bulk DNA sequencing data.

We obtained the strongest improvement when replacing the CVXOPT solver in Sapling (Qi and El-Kebir 2024) with *fastppm*-ADMM, where we obtained an average 406× improvement in runtime for n=50 mutations and an increase in method accuracy (Fig. 3a and Fig. 4, available as supplementary data at *Bioinformatics* online). Further, while Sapling was unable to infer phylogenies for instances beyond n=50 mutations within a 13 h time-limit, Sapling* successfully inferred phylogenies on all instances with n=500 mutations. The next largest improvement was found with CITUP*, which obtained a 5–10× improvement in runtime and an improved rate of successful termination within a 12-h time limit over CITUP while achieving similar accuracy in recovering the true phylogeny (Fig. 3b and Fig. 5, available as supplementary data at *Bioinformatics* online). Finally, Orchard* obtained a 3-5× improvement in runtime while maintaining similar accuracy in recovering the true phylogeny as compared to Orchard (Fig. 3c and Fig. 6, available as supplementary data at *Bioinformatics* online).

In summary, we find that incorporating *fastppm* in existing tumor phylogeny inference methods provides substantial improvements in runtime, supporting larger instances with little to no degradation in performance across all three methods.

### 4.3 Accurate phylogenetic reconstruction from low-coverage DNA sequencing data

To demonstrate the utility of directly modeling binomial loss rather than approximating the loss, we simulated 40 tumor phylogenies at 20× coverage and assessed how well the three top-performing methods, Sapling*, Orchard*, and Orchard, recovered the true phylogeny. We hypothesized that because Orchard and Orchard* rely on the $\ell_{2}$ loss for phylogenetic inference, while Sapling* directly models read counts via the binomial loss, the latter would produce more accurate phylogenies on such shallow sequencing data.

Confirming our hypothesis, we found that Sapling* outperformed Orchard and Orchard* in terms of both runtime and reconstruction accuracy on low coverage simulated bulk DNA sequencing data (Fig. 4). For example, on low-coverage data with n=50 mutations and m=50 samples, Orchard and Orchard* inferred phylogenies with a mean F1 score of 0.84 and 0.82, while the phylogenies inferred by Sapling* had a mean F1 score of 0.90. As the number of mutations increased, the trend held, with the performance of both methods degrading as the number of mutations increased (Fig. 4b and c). In terms of runtime, Sapling* was at least twice as fast as Orchard across all instances (Fig. 4a). However, without the improvement in runtime due to *fastppm*-ADMM, the runtime of Sapling would have been intractable for most use cases. In particular, processing instances with only n=250 mutations and m=50 samples would have taken an estimated 38 h using CVXOPT, in contrast to an average of 10 min with *fastppm*-ADMM.

### 4.4 Improved phylogenetic inference on a mouse model of colorectal cancer

Finally, we analyzed the patient-derived xenograft POP66 from a model of colorectal cancer in mouse, from which m=8 bulk samples underwent whole-exome sequencing at 50× coverage (Rehman *et al.* 2021). Following the analysis in the original publication, we excluded mutations that were contained in copy number impacted regions and used the copy number corrected read counts provided by (Rehman *et al.* 2021). After copy number correction, n=65 mutations were used for the analysis in (Rehman *et al.* 2021). To test the effect of directly modeling the binomial loss, rather than using the variant and total read counts directly we down-sampled the total read counts to 20× coverage (see Methods, available as supplementary data at *Bioinformatics* online). Then, we applied Orchard and Orchard*, which use the $\ell_{2}$ loss, and Sapling*, which uses *fastppm*-ADMM for the binomial loss, to the down-sampled data and compared the inferred frequencies to those inferred on the full data.

All methods completed tree inference in minutes, with Sapling* taking 3 min and 5 s, Orchard taking 4 min and 38 s, and Orchard* taking 1 min and 49 s when parallelized across a 16 core CPU. Notably, the original Sapling algorithm (which uses CVXOPT rather than *fastppm*-ADMM) would have taken over 22 h. The phylogeny inferred by Sapling* better recapitulated the down-sampled and original data compared to the phylogenies inferred by Orchard and Orchard* (Table 2). In more detail, we computed the $\ell_{2}$ error and the binomial loss for the frequency matrices $\hat{F}$ inferred by each method against the down-sampled and original data. For the $\ell_{2}$ error metric, the frequencies $\hat{F}$ inferred by Sapling* on the down-sampled data were 20% closer to the observed frequencies $\tilde{F}$ on the original data (Table 2). In terms of the binomial likelihood, the frequencies $\hat{F}$ inferred by Sapling* better explained (NLL: 10720.6) the observed variant reads as compared to those inferred by Orchard and Orchard* (NLL: 10793.5, 10790.9) (Table 2). Qualitatively, the phylogenies inferred by the three methods differed substantially (Figs 7–9 available as supplementary data at *Bioinformatics* online). For example, while the Orchard (Kulman *et al.* 2024) phylogeny had a truncal CNR1 mutation, the Sapling* phylogeny had a truncal KDR mutation (Figs 7–9, available as supplementary data at *Bioinformatics* online). However, the KDR and CNR1 mutations occurred relatively early in both phylogenies, suggesting a modest level of concordance.

**Table 2.** Analysis of POP66 derived xenograft data modeling colorectal cancer in mice (Rehman *et al.* 2021).^a^

Method	Metric	Metric Objective	
		Down-sampled	Original
Orchard^*^	$||\tilde{F}-\hat{F}|{|}_{F}^{2}$	4.183	3.092
Orchard	$||\tilde{F}-\hat{F}|{|}_{F}^{2}$	3.595	2.448
Sapling^*^	$||\tilde{F}-\hat{F}|{|}_{F}^{2}$	**3.421**	**2.181**
Orchard^*^	$-\text{log}\;P(V|T,\hat{F},D)$	2801.5	10790.9
Orchard	$-\text{log}\;P(V|T,\hat{F},D)$	2798.4	10793.5
Sapling^*^	$-\text{log}\;P(V|T,\hat{F},D)$	**2771.4**	**10720.6**

a The fit of the frequency matrices $\hat{F}$ inferred by Orchard*, Orchard (Kulman *et al.* 2024), and Sapling* to the original (resp. down-sampled) variant read count matrix V, total read count matrix D, and observed frequency matrix $\tilde{F}$. Bold values denote the minimum objective value across the methods.

These findings demonstrate that *fastppm* enables the application of more accurate loss functions, whose effect becomes especially prominent in shallow coverage settings.

## 5 Discussion

We introduced TSDDP, a new algorithmic technique capable of solving the Perfect Phylogeny Regression problem for arbitrary convex loss functions. Using TSDDP, we built *fastppm*, an easy-to-use tool which supports several convex loss functions, including the $\ell_{2}$, piecewise linear, binomial and beta-binomial loss. On both simulated and real data, we found that *fastppm* outperforms existing specialized and general-purpose algorithms, and that this translates to significant speedups when used in existing tumor phylogeny algorithms without loss of accuracy.

Even when the tumor phylogeny inference problem is highly under-determined (Qi *et al.* 2019), such as when the ratio of mutations-to-samples is large, and accurate reconstruction is impossible, we anticipate several applications of *fastppm*. For example, *fastppm* enables inference of mutation clusters by applying phylogenetically aware mutation clustering (Kulman *et al.* 2024, Schmidt and Raphael 2024) to mutation phylogenies built using *fastppm*. Using inferred mutation clusters, accurate reconstruction using *fastppm* becomes possible as the ratio of samples-to-clones is substantially larger. As another example, one could apply a summarization or consensus method, such as Sapling (Qi and El-Kebir 2024) or TuELiP (Guang *et al.* 2023), to a set of plausible phylogenies found by *fastppm*. As a final example, with cohort-level data (Jamal-Hanjani *et al.* 2017) one can find conserved evolutionary trajectories using a method such as MASTRO (Pellegrina and Vandin 2022) as phylogenies built using *fastppm* correctly capture many ancestral relationships.

As future work, the implementation of *fastppm* does not leverage the TSDDP algorithm’s ability to efficiently recompute the optimal objective upon slight perturbations to the tree topology. Second, while the PLA-*k* algorithm appears to construct an optimal piecewise linear approximation when the loss function is not-known beforehand, PLA-*k* could be improved by accounting for the structure of specific loss functions. Finally, we envision extending beyond the framework of maximum likelihood, instead computing the marginal likelihood with an integral over f respecting the (SC).

## Author contributions

Henri Schmidt (Conceptualization, Data curation, Formal analysis, Investigation, Software, Validation, Writing—original draft, Writing—review & editing), Yuanyuan Qi (Conceptualization, Data curation, Formal analysis, Software, Validation, Writing—–review & editing), Benjamin J. Raphael (Funding acquisition, Supervision), and Mohammed El-Kebir (Conceptualization, Formal analysis, Investigation, Software, Funding acquisition, Supervision, Validation, Writing—original draft, Writing—review & editing)

## Supplementary Material

btaf235_Supplementary_Data

## Data Availability

The data underlying this article are available in Supplementary material at Bioinformatics online, and in Github at https://github.com/elkebir-group/fastppm-data. Preprocessed colorectal data is publicly available on Github at https://github.com/morrislab/crc-dtp-ith-analysis/.

## References

[btaf235-B1] Addario-Berry L , Donderwinkel S. Random trees have height $O(\sqrt{n})$. Ann Probab 2024;52:1–2.

[btaf235-B2] Andersen MS, Dahl J, Vandenberghe L. CVXOPT: a python package for convex optimization. 2013.

[btaf235-B3] Boyd S. Distributed optimization and statistical learning via the alternating direction method of multipliers. FNT Mach Learn 2010;3:1–122.

[btaf235-B4] Boyd SP , Vandenberghe L. Convex Optimization. Cambridge, UK: Cambridge University Press, 2004, 215–87.

[btaf235-B5] Devroye L. Branching processes in the analysis of the heights of trees. Acta Inf 1987;24:1–2.

[btaf235-B6] Domahidi A, Chu E, Boyd S. ECOS: an SOCP solver for embedded systems. In: *Proceedings of the European Control Conference (ECC),* Zurich, Switzerland, 2013, 3071–6.

[btaf235-B7] El-Kebir M. SPhyR: tumor phylogeny estimation from single-cell sequencing data under loss and error. Bioinformatics 2018;34:i671–9. 30423070 10.1093/bioinformatics/bty589 PMC6153375

[btaf235-B8] El-Kebir M , Oesper L, Acheson-Field H et al Reconstruction of clonal trees and tumor composition from multi-sample sequencing data. Bioinformatics 2015;31:i62–70. 26072510 10.1093/bioinformatics/btv261 PMC4542783

[btaf235-B9] El-Kebir M , Satas G, Oesper L et al Inferring the mutational history of a tumor using multi-state perfect phylogeny mixtures. Cell Syst 2016;3:43–53. 27467246 10.1016/j.cels.2016.07.004

[btaf235-B10] Fernandez-Mateos J , Cresswell GD, Trahearn N et al Tumor evolution metrics predict recurrence beyond 10 years in locally advanced prostate cancer. Nat Cancer 2024;5:1334–51. 38997466 10.1038/s43018-024-00787-0 PMC11424488

[btaf235-B11] Funnell T , O'Flanagan CH, Williams MJ, IMAXT Consortium et al Single-cell genomic variation induced by mutational processes in cancer. Nature 2022;612:106–15. 36289342 10.1038/s41586-022-05249-0 PMC9712114

[btaf235-B12] Geißler B, Martin A, Morsi A *et al*. Using piecewise linear functions for solving MINLPs. In: *Mixed Integer Nonlinear Programming*. New York, NY: Springer, 2011, 287–314.

[btaf235-B13] Gerstung M , Jolly C, Leshchiner I et al, PCAWG Consortium. The evolutionary history of 2,658 cancers. Nature 2020;578:122–8. 32025013 10.1038/s41586-019-1907-7 PMC7054212

[btaf235-B14] Gillis S , Roth A. PyClone-VI: scalable inference of clonal population structures using whole genome data. BMC Bioinformatics 2020;21:571–16. 33302872 10.1186/s12859-020-03919-2 PMC7730797

[btaf235-B15] Goulart PJ , Chen Y. Clarabel: an interior-point solver for conic programs with quadratic objectives. *arXiv preprint arXiv:2405.12762*. 2024.

[btaf235-B16] Govek K , Sikes C, Oesper L. A consensus approach to infer tumor evolutionary histories. In: *ACM-BCB*, BCB ’18, New York, NY, USA. 2018, 63–72.

[btaf235-B17] Guang Z , Smith-Erb M, Oesper L. A weighted distance-based approach for deriving consensus tumor evolutionary trees. Bioinformatics 2023;39:i204–12. 37387177 10.1093/bioinformatics/btad230 PMC10311318

[btaf235-B18] Hanin B. Universal function approximation by deep neural nets with bounded width and relu activations. Mathematics 2019;7:992.

[btaf235-B19] Jamal-Hanjani M, Wilson GA, McGranahan N *et al*. Tracking the evolution of non–small-cell lung cancer. *New England J Med* 2017;376:2109–21. 10.1056/NEJMoa1616288 28445112

[btaf235-B20] Jia B, Ray S, Safavi S *et al*. Efficient projection onto the perfect phylogeny model. *Adv Neural Inf Process Syst* 2018;31:1–11.

[btaf235-B21] Kaufmann TL , Petkovic M, Watkins TBK et al MEDICC2: whole-genome doubling aware copy-number phylogenies for cancer evolution. Genome Biol 2022;23:241. 36376909 10.1186/s13059-022-02794-9 PMC9661799

[btaf235-B22] Kimura M. The number of heterozygous nucleotide sites maintained in a finite population due to steady flux of mutations. Genetics 1969;61:893–903. 5364968 10.1093/genetics/61.4.893 PMC1212250

[btaf235-B23] Kozlov A , Alves JM, Stamatakis A et al CellPhy: accurate and fast probabilistic inference of single-cell phylogenies from scDNA-seq data. Genome Biol 2022;23:37–0. 35081992 10.1186/s13059-021-02583-w PMC8790911

[btaf235-B24] Kulman E , Kuang R, Morris Q et al Orchard: building large cancer phylogenies using stochastic combinatorial search. PLoS Comput Biol 2024;20:e1012653. 39775053 10.1371/journal.pcbi.1012653 PMC11723595

[btaf235-B25] Laks E , McPherson A, Zahn H et al; CRUK IMAXT Grand Challenge Team. Clonal decomposition and DNA replication states defined by scaled single-cell genome sequencing. Cell 2019;179:1207–21.e22. 31730858 10.1016/j.cell.2019.10.026 PMC6912164

[btaf235-B26] Liu Y , Li XC, Rashidi Mehrabadi F et al Single-cell methylation sequencing data reveal succinct metastatic migration histories and tumor progression models. Genome Res 2023;33:1089–100. 37316351 10.1101/gr.277608.122 PMC10538489

[btaf235-B27] Malikic S , McPherson AW, Donmez N et al Clonality inference in multiple tumor samples using phylogeny. Bioinformatics 2015;31:1349–56. 25568283 10.1093/bioinformatics/btv003

[btaf235-B28] Minussi DC , Nicholson MD, Ye H et al Breast tumours maintain a reservoir of subclonal diversity during expansion. Nature 2021;592:302–8. 33762732 10.1038/s41586-021-03357-x PMC8049101

[btaf235-B29] MOSEK ApS. *The MOSEK Optimization Suite 10.2.13*. 2024.

[btaf235-B30] Myers MA , Satas G, Raphael BJ et al CALDER: inferring phylogenetic trees from longitudinal tumor samples. Cell Syst 2019;8:514–22.e5. 31229560 10.1016/j.cels.2019.05.010 PMC7263382

[btaf235-B31] Pellegrina L , Vandin F. Discovering significant evolutionary trajectories in cancer phylogenies. Bioinformatics 2022;38:ii49–55. 36124798 10.1093/bioinformatics/btac467

[btaf235-B32] Qi Y , El-Kebir M. Sapling: inferring and summarizing tumor phylogenies from bulk data using backbone trees. In: Pissis SP, Sung W-K (eds.), *24th International Workshop on Algorithms in Bioinformatics (WABI 2024)*, volume 312 of *Leibniz International Proceedings in Informatics (LIPIcs)*, Dagstuhl, Germany. Schloss Dagstuhl – Leibniz-Zentrum für Informatik. 2024, pages 7:1–7:19.

[btaf235-B33] Qi Y, Pradhan D, El-Kebir M. Implications of non-uniqueness in phylogenetic deconvolution of bulk DNA samples of tumors. Algorithm Mol Biol 2019;14:19. 10.1186/s13015-019-0155-6 PMC6719395 31497065

[btaf235-B34] Quinn JJ , Jones MG, Okimoto RA et al Single-cell lineages reveal the rates, routes, and drivers of metastasis in cancer xenografts. Science 2021;371:eabc1944. 33479121 10.1126/science.abc1944 PMC7983364

[btaf235-B35] Ray S, Jia B, Safavi S *et al*. Exact inference under the perfect phylogeny model. arXiv preprint arXiv:1908.08623. 2019.

[btaf235-B36] Rehman SK , Haynes J, Collignon E et al Colorectal cancer cells enter a diapause-like DTP state to survive chemotherapy. Cell 2021;184:226–42.e21. 33417860 10.1016/j.cell.2020.11.018 PMC8437243

[btaf235-B37] Rontsis N , Goulart P, Nakatsukasa Y et al Efficient semidefinite programming with approximate ADMM. J Optim Theory Appl 2022;192:292–320.

[btaf235-B38] Sankoff D , Rousseau P. Locating the vertices of a Steiner tree in an arbitrary metric space. Math Programm 1975;9:240–6.

[btaf235-B39] Satas G , Raphael BJ. Tumor phylogeny inference using tree-constrained importance sampling. Bioinformatics 2017;33:i152–60. 28882002 10.1093/bioinformatics/btx270 PMC5870673

[btaf235-B40] Satas G , Zaccaria S, El-Kebir M et al DeCiFering the elusive cancer cell fraction in tumor heterogeneity and evolution. Cell Syst 2021;12:1004–18.e10. 34416171 10.1016/j.cels.2021.07.006 PMC8542635

[btaf235-B41] Schmidt H , Raphael BJ. A regression based approach to phylogenetic reconstruction from multi-sample bulk DNA sequencing of tumors. PLoS Comput Biol 2024;20:e1012631. 39630782 10.1371/journal.pcbi.1012631 PMC11661639

[btaf235-B42] Schmidt H , Sashittal P, Raphael BJ et al A zero-agnostic model for copy number evolution in cancer. PLoS Comput Biol 2023;19:e1011590. 37943952 10.1371/journal.pcbi.1011590 PMC10662746

[btaf235-B43] Tseng P , Luo Z-Q. On computing the nested sums and infimal convolutions of convex piecewise-linear functions. J Algorithms 1996;21:240–66.

[btaf235-B44] Weber LL , Zhang C, Ochoa I et al Phertilizer: growing a clonal tree from ultra-low coverage single-cell DNA sequencing of tumors. PLoS Comput Biol 2023;19:e1011544. 37819942 10.1371/journal.pcbi.1011544 PMC10593221

[btaf235-B45] Wintersinger JA , Dobson SM, Kulman E et al Reconstructing complex cancer evolutionary histories from multiple bulk DNA samples using Pairtree. Blood Cancer Discov 2022;3:208–19. 35247876 10.1158/2643-3230.BCD-21-0092 PMC9780082

[btaf235-B46] Yang D , Jones MG, Naranjo S et al Lineage tracing reveals the phylodynamics, plasticity, and paths of tumor evolution. Cell 2022;185:1905–23.e25. 35523183 10.1016/j.cell.2022.04.015 PMC9452598

[btaf235-B47] Zhou Z , Xu B, Minn A et al DENDRO: genetic heterogeneity profiling and subclone detection by single-cell RNA sequencing. Genome Biol 2020;21:10. 31937348 10.1186/s13059-019-1922-x PMC6961311

