# Supplementary material for: Fast tumor phylogeny regression via tree-structured dual dynamic programming
Source: Bioinformatics. 2025 Jul 15;41(Suppl 1):i170–9. doi: 10.1093/bioinformatics/btaf235 (PMC12261417; doi:10.1093/bioinformatics/btaf235)
Supplement: btaf235_Supplementary_Data [file btaf235_supplementary_data.zip › fastppm_SuppText.pdf]

Supplementary Results

We first state two elementary observations which hold for all loss functions L_i and will be used in the following sections.

Observation 1. *$h_i = -L_i^*$ where $L_i^*(x) = \sup_f\{x \cdot f - L_i(f)\}$ is the convex conjugate of L_i.*

Observation 2. *J_i is concave.*

The case of quadratic loss

In the case of a weighted quadratic loss we have $L_i(f_i) = w_i(f_i - \tilde{f}_i)^2$, where $\tilde{f}_i$ denotes the observed frequencies and $w_i \geq 0$ denotes the weight assigned to the ith frequency. Since $h_i = -L_i^*$, where L_i^* is the convex conjugate of the quadratic loss L_i, we have $h_i(\alpha_i) = -\left(\frac{\alpha_i^2}{4w_i} + \tilde{f}_i\alpha_i\right)$. When i is a leaf, $J_i(\gamma) = \max_{\alpha_i \geq 0}\{h_i(\alpha_i - \gamma)\}$, and by characterizing the maximizer in terms of the derivative, we have

$$J_i(\gamma) = \begin{cases} \gamma\tilde{f}_i - \frac{\gamma^2}{4w_i} & \text{if } \gamma \leq 2w_i\tilde{f}_i, \\ w_i\tilde{f}_i^2 & \text{otherwise.} \end{cases} \tag{8}$$

In this case, J_i has a piecewise linear derivative and as we will soon show, J_i always has a piecewise linear derivative. Consequently, we fix (i) the computational representation $\mathcal{R}(J_i)$ to consist of the value $J_i(0)$ along with the slopes, intercepts, and breakpoints of the derivative J_i'. In this representation, the value $J_i(0)$ is stored as a scalar, while the slopes, breakpoints and intercepts of J_i' are stored in three one-dimensional arrays sorted in ascending order of the breakpoints. To compute the representation (ii) of the leaves, it suffices to use (8) to obtain the representation of J_i, taking $\mathcal{O}(1)$ time since J_i' has only a single breakpoint.

It remains to show that J_i always has a piecewise linear derivative and describe how to compute the representation $\mathcal{R}(J_i)$ at an internal node i. To do this, we assume that the inductive hypothesis (i.e. J_i has a piecewise linear derivative) holds for all descendants of node i. By the base case given in (8), the inductive hypothesis holds for the deepest, non-leaf node, allowing us to propagate the claim up the tree.

Assuming that for all descendants j of node i, the representations $\mathcal{R}(J_j)$ are available and J_j' is a piecewise linear function, we first characterize the maximizer in $J_i(\gamma)$,

$$\alpha_i^*(\gamma) := \operatorname*{arg\,max}_{\alpha_i \geq 0}\left\{h_i(\alpha_i - \gamma) + \sum_{j \in \delta(i)} J_j(\alpha_i)\right\},$$

where we let arg max denote the unique, left most maximizer. Since the inner term is concave by (Observation 2), it follows that

$$\alpha_i^*(\gamma) = \inf\left\{\alpha_i \geq 0 : h_i'(\alpha_i - \gamma) + \sum_{j \in \delta(i)} J_j'(\alpha_i) \leq 0\right\}, \tag{9}$$

where we take the infimum of the empty set to be 0. The reason for studying the arg max rather than the max is that the derivative of a (piecewise) quadratic function is (piecewise) linear, and consequently easier to work with.

Next, assuming that the derivatives J_j', $j \in \delta(i)$, have at most k_j pieces with $k = \sum_{j\in\delta(i)} k_j$, we write the sum

$$g_i(\alpha_i) := \sum_{j \in \delta(i)} J_j'(\alpha_i) = (c_0 + m_0\alpha_i) \cdot \mathbb{1}(\alpha_i \in (b_0, b_1]) + \sum_{j=1}^{k}(c_j + m_j(\alpha_i - b_j)) \cdot \mathbb{1}(\alpha_i \in (b_j, b_{j+1}]) \tag{10}$$

where $-\infty = b_0 < b_1 < \ldots < b_k < b_{k+1} = +\infty$ are the k distinct breakpoints of the piecewise linear functions J_j'. As h_i' is affine, we have that

$$\begin{aligned} h_i'(\alpha_i - \gamma) + g(\alpha_i) &= -(\tilde{f}_i + \frac{1}{2w_i}(\alpha_i - \gamma)) + (c_0 + m_0\alpha_i) \cdot \mathbb{1}(\alpha_i \in (b_0, b_1]) + \sum_{j=1}^{k}(c_j + m_j(\alpha_i - b_j)) \cdot \mathbb{1}(\alpha_i \in (b_j, b_{j+1}]) \\ &= (\frac{\gamma}{2w_i} - \tilde{f}_i) + (c_0' + m_0'\alpha_i) \cdot \mathbb{1}(\alpha_i \in (b_0, b_1]) + \sum_{j=1}^{k}\left[c_j' + m_j'(\alpha_i - b_j)\right] \cdot \mathbb{1}(\alpha_i \in (b_j, b_{j+1}]), \end{aligned}$$

where $m_j' = m_j - \frac{1}{2w_i}$ for $j = 0, \ldots, k$, $c_0' = c_0$, and $c_j' = c_j - \frac{b_j}{2w_i}$ for $j = 1, \ldots, k$. Then, $\alpha_i^*(\gamma)$ is the smallest point $\alpha_i \geq 0$ such that

$$\alpha_i \in (b_j, b_{j+1}] \quad \text{and} \quad c_j' + m_j'(\alpha_i - b_j') \leq \tilde{f}_i - \frac{\gamma}{2w_i}, \tag{11}$$

where $b_0' = 0, b_1' = b_1, \ldots, b_{k+1}' = b_{k+1}$. We can now use this relation to show that α_i^* is linear on certain pieces, formalized as follows.

Lemma 1. *If* $c'_{j+1} \leq \tilde{f}_i - \frac{\gamma}{2w_i} \leq c'_j$, *then* $b_j \leq \alpha^*_i(\gamma) \leq b_{j+1}$.

Proof If $\alpha_i < b_j$, we have $\alpha_i \in (b_l, b_{l+1}]$ for some $l < j$. However, since $c'_1 > c'_2 > \dots$ from the fact that $g_i(\alpha)$ is decreasing (as J_i is concave), this implies that $c_l + m_l(\alpha_i - b'_l) > c'_j$. Consequently, α_i could not satisfy (11) if $\alpha_i < b_j$. In the other direction, since $\alpha_i = b_{j+1}$ satisfies (11), no $\alpha_i > b_{j+1}$ can be the smallest such α_i satisfying (11). This completes the proof. □

Since $h'_i(\alpha_i - \gamma) + g_i(\alpha_i)$ is linear on each piece $[b_j, b_{j+1}]$, we derive the following explicit form of $\alpha^*_i(\gamma)$, completing our characterization of α^*_i.

Lemma 2. *Let* $z_j = 2w_i(\tilde{f}_i - c'_j)$. *Then,*

$$\alpha^*_i(\gamma) = \max\left\{\frac{\tilde{f}_i - \frac{\gamma}{2w_i} - c'_j}{m'_j} + b'_j, 0\right\} \quad \textit{for} \quad z_j \leq \gamma \leq z_{j+1}, \tag{12}$$

Proof Suppose $z_j \leq \gamma \leq z_{j+1}$. Then, if $\alpha^*_i(\gamma) > 0$, by Lemma 1 we have that

$$\begin{aligned}\alpha^*_i(\gamma) &= \inf\{\alpha_i \geq 0 : c'_j + m'_j(\alpha_i - b'_j) \leq \tilde{f}_i - \frac{\gamma}{2w_i}\} \\ &= \inf\{\alpha_i \geq 0 : c'_j + m'_j(\alpha_i - b'_j) = \tilde{f}_i - \frac{\gamma}{2w_i}\}\end{aligned}$$

where the first equality follows from the assumption that $z_j \leq \gamma \leq z_{j+1}$ and the second from the linearity of $h_i(\alpha_i - \gamma) + g_i(\alpha_i)$ when $\alpha_i \in [b_j, b_{j+1}]$. Solving the equality for α^*_i and taking care of non-negativity completes the proof. □

Now, we are ready to obtain an explicit form for $J'_i(\gamma)$. In particular, we have the following form for J'_i.

Lemma 3. *Let* $z_j = 2w_i(\tilde{f}_i - c'_j)$ *and suppose* $\gamma \in [z_j, z_{j+1}]$ *with* $b_j \geq 0$. *Then,*

$$J'_i(\gamma) = \frac{m_j(2w_i\tilde{f}_i + b_j - \gamma) - c_j}{2w_i m_j - 1}. \tag{13}$$

Further, J'_i *is piecewise linear.*

Proof Using the chain rule for subgradients, we have that

$$J'_i(\gamma) = h'_i(\alpha^*_i(\gamma) - \gamma)[\alpha^*_i(\gamma) - \gamma]' + g_i(\alpha^*_i(\gamma))[\alpha^*_i(\gamma)]'. \tag{14}$$

Since h'_i is affine, an explicit expression for the first term is straightforward to compute. Next, we note that by Lemma 1, α^*_i is piecewise linear. Since g_i is also piecewise linear, this implies that J'_i is piecewise linear with a finite number of pieces.

Next, note that α^*_i is monotonically increasing: to see this, observe $m'_j \leq 0$ by the concavity of J_i and h'_i, and use the expression given in Lemma 1. A quick computation then reveals $\alpha^*_i(z_j) = b_j$, provided $b_j \geq 0$, implying $\alpha^*_i([z_j, z_{j+1}]) = [b_j, b_{j+1}]$. Using (14), it then follows that J'_i is also linear when $\gamma \in [z_j, z_{j+1}]$, since then $\alpha_i(\gamma) \in [b_j, b_{j+1}]$. Taking advantage of this linearity, plugging in (12) to (14), and performing algebraic manipulations verified using the computer algebra system Mathematica v14.1, we obtain (13). □

Setting $l = \min\{j : b_j \geq 0\}$, we have obtained an explicit form for J'_i for all $\gamma \geq z_l$. In particular, from z_l to ∞, $J'_i(\gamma)$ is piecewise linear with breakpoints $z_l \leq \dots \leq z_k$ and slopes and intercepts given by (13). Thus, in $\mathcal{O}(k)$ time we are able to compute the slopes, intercepts and breakpoints of $J'_i(\gamma)$ for $\gamma \geq z_l$, nearly completing (iii) of the TDSPP algorithm. To the left of z_l, there can be at most one additional breakpoint, which is somewhere on the interval $[b_{l-1}, b_l]$. By a similar line of reasoning, we can find this breakpoint, called x, using Lemma 1. Clearly, for $\gamma \in [x, b_l]$, $J'_i(\gamma)$ is again linear, with its slope and intercept easily computed. Since for all $\gamma \leq x$, $J'_i(\gamma) = 0$, we have completely described the slopes and intercepts of J'_i using only $\mathcal{O}(k)$ time. Further, we have shown that the total number of distinct breakpoints in J'_i is bounded by k, the total number of breakpoints in the functions J'_j, $j = 1, \dots, k$. We are now ready to complete the proof of Theorem 2.

Proof of Theorem 2. For the base case (ii) of a tree with a single node it suffices to compute the representation of a single leaf, which by (8) takes $\mathcal{O}(1)$ time.

For the inductive step (iii), consider a tree $\mathcal{T}$ with n nodes and $l = |\delta(r)|$ subtrees $\mathcal{T}_{i_1}, \ldots, \mathcal{T}_{i_l}$ rooted at the children $\delta(r) = \{i_1, \ldots, i_l\}$ of the root vertex r of $\mathcal{T}$ and suppose the representations $\mathcal{R}(J_{i_1}), \ldots, \mathcal{R}(J_{i_l})$ have already been computed. To compute $\mathcal{R}(J_i)$, we first compute $g_i = J'_{i_1} + \cdots + J'_{i_l}$, which consists of the summation of l piecewise linear functions with a total of $n-1$ breakpoints, following the preceding arguments. This summation can be performed in at most $C_1 n \log(l)$ time for some constant C_1 by recursively merging the l arrays of sorted breakpoints in the representations $\mathcal{R}(J_{i_j})$ while keeping track of the associated slopes. Then, using Lemma 3 and the preceding arguments, we compute J'_i using an additional $C_2 n$ time for some constant C_2. To compute the intercept $J_i(0)$ also takes $C_2 n$ time, using the equality $J_i(0) = h_i(\alpha_i^*(0)) + J_{i_1}(\alpha_i^*(0)) + \cdots + J_{i_R}(\alpha_i^*(0))$.

Adding the time taken at each node, it takes a total of T time to compute the representation $\mathcal{R}(J_r)$ of the root vertex:

$$T = \sum_{v \in V(\mathcal{T})} (2C_2 S_v + C_1 S_v \log R_v), \tag{15}$$

where $S_v = |V(\mathcal{T}_v)|$ is the size of the subtree rooted at v and $R_v = |\delta(v)|$ is the number of children of vertex v. We bound T using Holder's inequality in two different ways. First, we have

$$\begin{aligned}
T &= \sum_{v \in V(\mathcal{T})} (2C_2 S_v + C_1 S_v \log R_v) \\
&\leq 2C_2 \cdot \sum_{v \in V(\mathcal{T})} n + C_1 \cdot \sum_{v \in V(\mathcal{T})} S_v \log R_v && \text{since } S_v \leq n, \\
&\leq 2C_2 n^2 + C_1 (\max_{v \in V(\mathcal{T})} S_v) \cdot (\sum_{v \in V(\mathcal{T})} \log R_v) && \text{using Holder's inequality,} \\
&\leq 2C_2 n^2 + C_1 n^2 && \text{since } \sum_{v \in V(\mathcal{T})} R_v = n-1 \text{ and } S_v \leq n,
\end{aligned}$$

yielding a runtime bound of $\mathcal{O}(n^2)$. Second, let $S = \frac{1}{n} \sum_{v \in V(\mathcal{T})} S_v$, we then have

$$\begin{aligned}
T &= \sum_{v \in V(\mathcal{T})} (2C_2 S_v + C_1 S_v \log R_v) \\
&= 2C_2 nS + C_1 \sum_{v \in V(\mathcal{T})} S_v \log R_v && \text{by definition of } S, \\
&\leq 2C_2 nS + C_1 (\sum_{v \in V(\mathcal{T})} S_v) \cdot (\max_{v \in V(\mathcal{T})} \log R_v) && \text{using Holder's inequality,} \\
&= 2C_2 nS + C_1 nS \cdot (\max_{v \in V(\mathcal{T})} \log R_v).
\end{aligned}$$

This yields the second runtime bound of $\mathcal{O}(nS \log R) = \mathcal{O}(Sn \log R)$. However, it is well known that $S = \frac{1}{n} \sum_{v \in V(\mathcal{T})} (1 + d_v) = 1 + \bar{d}$ where d_v is the distance of v from the root to v. This is because in computing $\sum_{v \in V(\mathcal{T})} S_v$, each node v is counted a total of $d_v + 1$ times, once for each ancestor of v. Consequently, we obtain a third bound of $\mathcal{O}(n\bar{d} \log R)$, completing the proof. □

The case of convex piecewise linear loss

In the case of a convex, piecewise linear loss function with k breakpoints, we have

$$L_i(f_i) = \sum_{j=1}^{k} (c_{i,j} + m_{i,j} f_i) \mathbb{1}(f_i \in (x_j, x_{j+1}]), \tag{16}$$

where $-\infty = x_0 < x_1 < x_2 < \ldots < x_{k+1} = +\infty$ are the k distinct breakpoints of L_i. To design our TSDDP algorithm for this class of loss functions, we show that steps (i)-(iv) consist solely of the operations of addition, conjugation, and infimal convolution (Boyd and Vandenberghe, 2004) of convex piecewise linear functions, for which specialized algorithms and data structures have been developed (Tseng and Luo, 1996).

Specifically, given two convex functions $f, g : \mathbb{R} \to \mathbb{R} \cup \{\pm\infty\}$, we denote by $f + g$ the sum, by $f \,\square\, g$ the *infimal convolution*, and by f^* the *convex conjugate* which are defined pointwise as,

$$(f+g)(x) = f(x) + g(x), \quad (f \,\square\, g)(x) = \inf_y \{f(y) + g(x-y)\}, \quad \text{and} \quad f^*(x) = \sup_y \{xy - f(y)\}.$$

Importantly, we note that the infimal convolution is related to conjugation through the identity

$$f \,\square\, g = (f^* + g^*)^*. \tag{17}$$

Under this notation, it follows that both steps (ii) and (iii) of the TSDDP algorithm can be written in terms of the three aforementioned operations. Namely, for a vertex i, we have that

$$\begin{aligned}
J_i(\gamma) &= \sup_{\alpha_i \ge 0} \{h_i(\alpha_i - \gamma) + \sum_{j\in\delta(i)} J_j(\alpha_i)\} && (18)\\
&= \sup_{\alpha_i}\{h_i(\alpha_i - \gamma) + \sum_{j\in\delta(i)} J_j(\alpha_i) - I(\alpha_i)\} \qquad \text{by defining } I(\alpha_i) := \begin{cases}\infty & \text{if } \alpha_i \le 0,\\ 0 & \text{otherwise.}\end{cases} && (19)\\
&= -\inf_{\alpha_i}\{-h_i(\alpha_i - \gamma) - \sum_{j\in\delta(i)} J_j(\alpha_i) + I(\alpha_i)\} \qquad \text{using } \sup_x f(x) = -\inf_x\{-f(x)\}, && (20)\\
&= -((I - \sum_{j\in\delta(i)} J_j) \,\square\, (-h_i)) \qquad \text{by definition of } \square, && (21)\\
&= -((I - \sum_{j\in\delta(i)} J_j) \,\square\, L_i^*) \qquad \text{by Observation 1.} && (22)
\end{aligned}$$

That is, computing the representation $\mathcal{R}(J_i)$ is equivalent to computing the representation after performing the operations of summation and conjugation, followed by infimal convolution.

In more detail, at a leaf vertex i, it follows from (22) that $J_i = -(I \,\square\, L_i^*) = -(I^* + L_i)^*$, which is the negation of the conjugation of a convex, piecewise linear function. From Observation 1 in (Tseng and Luo, 1996), it follows J_i is concave and piecewise linear with k breakpoints, where the breakpoints of L_i become the slopes of J_i and the slopes of L_i become the breakpoints of J_i. Inductively assuming that J_j are concave and piecewise linear for all children $j \in \delta(i)$, we have that $I - \sum_{j\in\delta(i)} J_j$ is convex and piecewise linear. Again applying Observation 1 from (Tseng and Luo, 1996), it follows that $J_i = -((I - \sum_{j\in\delta(i)} J_j) \,\square\, L_i^*)$ is concave and piecewise linear.

Since J_i is a concave, piecewise linear function, we fix (i) the representation $\mathcal{R}(J_i)$ to consist of the slopes, breakpoints, and intercept of J_i. However, rather than naïvely storing the slopes and breakpoints in an array or a linked list, we use the representation described in (Tseng and Luo, 1996) which represents the breakpoints and slopes in a balanced binary search tree by storing the *difference* in the slopes and breakpoints on the nodes of the tree. Indeed, using such a representation, (Tseng and Luo, 1996) proves (see Proposition 6.1) that obtaining the representation of the sum of l concave, piecewise linear functions with $k_1 \le \ldots \le k_l$ breakpoints, $K = \sum_{i=1}^{l-1} k_i$, takes $\mathcal{O}(K \log^2 K)$ time. Similarly, (Tseng and Luo, 1996) proves that obtaining the representation of the infimal convolution of two piecewise linear functions with K and K' breakpoints takes $\mathcal{O}(\min\{K, K'\} \log^2(K + K'))$ time.

We are now ready to prove Theorem 3.

Proof of Theorem 3. Suppose inductively that for all trees $\mathcal{T}$ of size less than $n' < n$ we can compute the representation $\mathcal{R}(J_{r(\mathcal{T})})$ in $\mathcal{O}(n' \log^2(n'k))$ time. This holds for the base case (ii) of a tree with a single node, since it then suffices to compute the representation of a single leaf, which by the preceding arguments takes $\mathcal{O}(k \log^2(k))$ time.

Next, consider a tree $\mathcal{T}$ with n nodes and $R = |\delta(r)|$ subtrees $\mathcal{T}_{i_1}, \ldots, \mathcal{T}_{i_R}$ rooted at the children $\delta(r) = \{i_1, \ldots, i_R\}$ of the root vertex r of $\mathcal{T}$. By the inductive hypothesis, we can compute the representations $\mathcal{R}(J_{i_1}), \ldots, \mathcal{R}(J_{i_R})$ in $\mathcal{O}((n-1)k \log^2(nk))$ time. By the preceding arguments, obtaining the representation $\mathcal{R}(J_r)$ consists of first obtaining the representation of $I - (J_{i_1} + \cdots + J_{i_R})$, which takes $\mathcal{O}((n-1)k \log^2(nk))$ time as the total number of breakpoints in $I, J_{i_1}, \ldots, J_{i_R}$ is bounded by $(n-1)k+1$. Computing the representation of the convolution $(I - (J_{i_1} + \cdots + J_r)) \,\square\, L_r^*$ requires only an additional $\mathcal{O}(k \log^2(nk))$ time as L_r^* only has k pieces. Consequently, the time to compute the representation $\mathcal{R}(J_r)$ is $\mathcal{O}(nk \log^2(nk))$ in total.

Observing that a concave, piecewise linear function must be maximized at a breakpoint, we solve the one-dimensional optimization problem (iv) in $\mathcal{O}(nk)$ time by scanning over the breakpoints. This completes the proof. □

Convexity of the negative beta-binomial log-likelihood

The beta-binomial distribution, $\mathrm{BetaBin}(d, \alpha, \beta)$, is a compound distribution described in two steps. First, the binomial proportion p is drawn from a beta distribution parameterized by shape parameters α, β, i.e. $p \sim \mathrm{Beta}(\alpha, \beta)$. In our setting, the mean of this beta distribution corresponds to the frequency f, i.e. $f = \alpha/(\alpha + \beta)$. Second, the number of successes, or in our case the number of variant reads v, are drawn from a binomial distribution with d trials and success probability p, i.e. $v \sim \mathrm{Bin}(d, p)$. The probability mass function then equals:

$$\mathbb{P}(v \mid \alpha, \beta, d) = \frac{\Gamma(d+1)\Gamma(v+\alpha)\Gamma(d-v+\beta)\Gamma(\alpha+\beta)}{\Gamma(v+1)\Gamma(d+\alpha+\beta)\Gamma(d-v+1)\Gamma(\alpha)\Gamma(\beta)} \tag{23}$$

where $\Gamma(x)$ is the gamma function.

We use a re-parametrization where, rather than the two shape parameters α, β, we are given a precision parameter $s = \alpha + \beta$. In our setting, in addition to s, we are given the total number d of reads (or trials) and the variant number

v of reads (or successes). The frequency f (or mean of the beta distribution) is the parameter of interest in our setting. Since $s = \alpha + \beta$ and $f = \alpha/(\alpha + \beta) = \alpha/s$, we have $\alpha = fs$ and $\beta = s - fs$, which allows us to rewrite (23) as

$$\mathbb{P}(v \mid f, s, d) = \frac{\Gamma(d+1)\Gamma(v+fs)\Gamma(d-v+s-fs)\Gamma(s)}{\Gamma(v+1)\Gamma(d+s)\Gamma(d-v+1)\Gamma(fs)\Gamma(s-fs)}. \tag{24}$$

Ignoring constant terms with respect to the parameter f, the negative log-likelihood is up to an additive constant-factor then equal to:

$$L(f \mid v, d) \propto -\log \mathbb{P}(v \mid f, s, d) \propto \log \Gamma(fs) - \log \Gamma(v + fs) + \log \Gamma(s - fs) - \log \Gamma(d - v + s - fs). \tag{25}$$

Convexity of $L(f \mid v, d)$ with respect to f can be shown using the monotonicity (decreasing) of the trigamma function (i.e. $\frac{d^2}{dz^2} \ln \Gamma(z)$).

Supplementary Methods

Progressive Piecewise Linear Approximation (PPLA)

PPLA(k, ρ, τ) takes as input three parameters: (i) the number $k \in \mathbb{N}$ of segments, (ii) a multiplicative factor $\rho \in [0, 1]$ by which to adjust the search range, and (iii) a threshold parameter $\tau \in [0, 1]$ used to terminate the procedure. The algorithm is as follows.

1. Set bounds $[\mathbf{f}^-, \mathbf{f}^+] := [\mathbf{0}, \mathbf{1}]$ and range $r := 1$.
2. Obtain frequencies $\mathbf{f}$ by solving k-PLA with frequency lower bounds $\mathbf{f}^-$ and upper bounds $\mathbf{f}^+$.
3. Update range $r \leftarrow r \cdot \rho$.
4. For each mutation $i \in [n]$:
   i. Update frequency lower bound $f_i^- \leftarrow f_i - r/2$.
   ii. Update frequency upper bound $f_i^+ \leftarrow f_i + r/2$.
5. If range $r > \tau$, go to Step 2, otherwise return $\mathbf{f}$.

Since the range r of PPLA(k, ρ, τ) shrinks by the constant factor ρ at every iteration of the algorithm, PPLA(k, ρ, τ) performs $\frac{\log \tau}{\log \rho}$ calls to k-PLA, yielding a total time complexity of $\mathcal{O}(nk \log^2 nk \cdot \log \tau \cdot \log^{-1} \rho)$. Further, since PPLA$(k, \rho, \tau)$ improves the loss at every iteration, it enjoys the same convergence properties as k-PLA (Theorem 4). Practically, however, we have observed that PPLA(k, ρ, τ) finds more accurate solutions than k-PLA, albeit at the cost of increased runtime.

Simulation details

Each simulation was defined by a tuple of four parameters (n, m, c, r), as described below.

n: the number of mutations/nodes in $\mathcal{T}$.
m: the number of samples.
c: the expected number of total reads or *coverage*.
r: the random seed.

The output of each simulation is a clonal tree $\mathcal{T}$ on n mutations, a n-by-n clonal matrix $\mathbf{B}$, a m-by-n variant read count matrix $\mathbf{V}$, a m-by-n- total read count matrix $\mathbf{D}$, a m-by-n frequency matrix $\mathbf{F}$, and an observed frequency matrix $\widehat{\mathbf{F}}$.

To construct a simulated instance for a fixed set of parameters (n, m, c, r), we first uniformly at random sampled, using Wilson's algorithm (Wilson, 1996), a clonal tree $\mathcal{T}$ with n mutations and constructed the associated clonal matrix $\mathbf{B}$. Then, for each of the m samples, we sampled a usage vector $\mathbf{u}_i^T$ from a Dirichlet distribution, stacked them into a usage matrix $\mathbf{U} = [\mathbf{u}_i]$, and fixed the frequency matrix as $\mathbf{F} = \mathbf{UB}$. To generate the observed read counts, we sampled the total number d_{ij} of reads for the jth mutation in the ith sample using $d_{ij} \sim \text{Poisson}(c)$. Then, using the total read counts, the variant reads were sampled using $v_{ij} \sim \text{Binomial}(d_{ij}, f_{ij})$. To obtain variant and total read count matrices, we set $\mathbf{V} = [v_{ij}]$ and $\mathbf{D} = [d_{ij}]$. The observed frequency matrix was set to $\widehat{\mathbf{F}} = [v_{ij}/d_{ij}]$.

Executing and evaluating regression algorithms

We evaluated five existing regression algorithms for the ℓ_2 and binomial negative log-likelihood loss against *fastppm* on 90 simulated tumor phylogenies containing $n \in \{100, 500, 1000, 2000, 3000, 4000\}$ mutations and a read coverage of $c \in \{30, 100, 1000\}$ across $r \in \{1, 2, 3, 4, 5\}$ random number generator seeds. For the ℓ_2 loss, we evaluated the specialized regression algorithm *projectppm* (Jia et al., 2018). In addition, we evaluated state-of-the-art convex optimization solvers CVXOPT (Andersen et al., 2013), ECOS (Domahidi et al., 2013), Mosek (MOSEK ApS, 2024), and Clarabel (Goulart and Chen, 2024) on both the ℓ_2 and binomial log-likehood loss.

To build *projectppm* (Jia et al., 2018), we compiled the software using GCC with optimization flags `-O3` and `-ffast-math`. Then, to execute the software, we passed in the observed frequency matrix $\widehat{\mathbf{F}}$ and the ground truth clonal tree $\mathcal{T}$ to the solver. Additionally, we specified that the solver only output the optimal objective value and not the inferred usage and frequency matrices. Finally, to time the software, we used the Linux tool `/usr/bin/time`, which provided a sufficiently high precision clock to benchmark the solver runtime.

To evaluate conic optimization solvers ECOS (Domahidi et al., 2013), Mosek (MOSEK ApS, 2024), and Clarabel (Goulart and Chen, 2024), we accessed the solvers through the Python convex optimization interface CVXPY (Diamond and Boyd, 2016). As the objective was trivially separable across the m samples, to ensure a fair comparison, we passed each sample one at a time using CVXPY's *Parameter* object. Then, for each sample p, we setup the following optimization

problem in CVXPY:

$$\begin{aligned} \min \quad & \sum_{j=1}^{n} L_j(f_{pj}) \\ \text{s.t.} \quad & \mathbf{f}_p^T = \mathbf{u_p^T B}, \\ & \mathbf{u_p}^T \mathbb{1} \leq 1, \\ & \mathbf{u_p} \geq 0, \\ & \mathbf{f_p} \leq 1 - \epsilon, \\ & \mathbf{f_p} \geq \epsilon. \end{aligned}$$

For the binomial log-likelihood loss, we fixed $\epsilon = 10^{-5}$ to avoid numerical stability issues which persisted across all solvers, while we set $\epsilon = 0$ for the ℓ_2 loss. For the binomial log-likelihood loss, we set $L_p(f_{pj}) = -v_{pj} \log(f_{pj}) - (d_{pj} - v_{pj}) \log(1 - f_{pj})$. For the ℓ_2 loss, we set $L_j(f_{pj}) = (f_{pj} - \hat{f}_{pj})^2$. To benchmark the solvers, we recorded the solve time as provided by the solver and ignored the time spent building the model and the time spent in CVXPY's interface. Since each sample was processed independently, we summed the runtime and objective across all m samples to obtain the final runtime and objective.

To evaluate convex optimization solver CVXOPT (Andersen et al., 2013), we used the non-linear objective convex optimization solver `cvxopt.solvers.cp` provided through the Python interface. To use the solver, we passed in a helper function which computes the objective $L(\mathbf{F})$, the gradient $\nabla L(\mathbf{F})$, and the Hessian matrix $\nabla^2 L(\mathbf{F})$ of the objective

$$L(\mathbf{F}) = \sum_{p=1}^{m} \sum_{j=1}^{n} L_j(f_{pj}),$$

with respect to the flattened frequency matrix $\operatorname{vec} \mathbf{F}$. Since the Hessian matrix is diagonal, we returned a sparse representation of the nm-by-nm Hessian matrix. In total, computing the objective, gradient, and Hessian matrix took $\mathcal{O}(nm)$ time. To benchmark the solver, we used the runtime and objective value reported by the solver.

All solvers were ran with default parameters, excepting an increased number of maximum iterations for the Clarabel and ECOS solvers to improve the solvers' success rate. An execution of a solver was called successful if for all m samples, the solver successfully terminated and emitted a finite objective value.

Executing and evaluating tree inference algorithms

To evaluate the resulting six methods, we simulated tumor phylogenies using an identical procedure as in Simulation details, but varied the parameter settings to fit each method. For Sapling and Orchard, which progressively grow clonal trees, we simulated 120 tumor phylogenies with $n \in \{50, 100, 250, 500\}$ mutations, $m \in \{50, 100\}$ samples, and $c \in \{30, 100, 1000\}$ read coverage across $s \in \{1, \ldots, 5\}$ random number generator seeds. For CITUP, which exhaustively enumerates all rooted, unlabeled trees with n nodes, we simulated 180 tumor phylogenies with $n \in \{3, 5, 10\}$ mutations, $m \in \{3, 5\}$ samples, and $c \in \{30, 100, 1000\}$ read coverage across $s \in \{1, \ldots, 5\}$ random number generator seeds. When running the six methods, we provided Sapling and Sapling* with a single core and four GB of memory, Orchard and Orchard* with eight cores, and four GB of memory and CITUP and CITUP* with sixteen cores and four GB of memory on 2.4 GhZ CPUs.

For each method and simulated instance, we measured the wall-clock runtime and the accuracy in recovering the ground truth tumor phylogeny, quantified in terms of the normalized parent-child distance, the normalized ancestor-descendant distance, the false positive rate, the false negative rate, and the F1 score. The (normalized) parent-child distance (Qi and El-Kebir, 2024) between two directed graphs $\mathcal{T}_1, \mathcal{T}_2$ on the same vertex set $V(\mathcal{T}_1) = V(\mathcal{T}_2)$ is the normalized symmetric difference of their edge sets. Namely,

$$d_{\mathrm{PC}}(\mathcal{T}_1, \mathcal{T}_2) := \frac{|E(\mathcal{T}_1) \triangle E(\mathcal{T}_2)|}{|E(\mathcal{T}_1) \cup E(\mathcal{T}_2)|}. \tag{26}$$

The (normalized) ancestor-descendant distance (Govek et al., 2018) is then defined as the parent-child distance between the transitive closures $\mathrm{cl}(\mathcal{T}_1), \mathrm{cl}(\mathcal{T}_2)$. Namely,

$$d_{\mathrm{AD}}(\mathcal{T}_1, \mathcal{T}_2) := d_{\mathrm{PC}}(\mathrm{cl}(\mathcal{T}_1), \mathrm{cl}(\mathcal{T}_2)). \tag{27}$$

Both metrics take values in the interval $[0, 1]$, with $d(\mathcal{T}_1, \mathcal{T}_2) = 0$ if and only if $\mathcal{T}_1 = \mathcal{T}_2$.

To obtain the false positive rate, false negative rate, and F1 score, we view phylogenetic inference as a binary classification task where the goal is to predict the presence or absence of edges in the transitive closure $\mathrm{cl}(\mathcal{T}_1)$ of the ground truth tree $\mathcal{T}_1$. Specifically, we call $(i, j) \in E(\mathrm{cl}(\mathcal{T}_2))$ a *true positive* if $(i, j) \in E(\mathrm{cl}(\mathcal{T}_1))$ and *false positive* if $(i, j) \notin E(\mathrm{cl}(\mathcal{T}_1))$. Similarly, we call $(i, j) \notin E(\mathrm{cl}(\mathcal{T}_2))$ a *true negative* if $(i, j) \notin E(\mathrm{cl}(\mathcal{T}_1))$ and *false negative* if $(i, j) \in E(\mathrm{cl}(\mathcal{T}_1))$. Counting the number of false positives, false negatives, true positives, and true negatives in the inferred tree $\mathcal{T}_2$, the false positive rate, false negative rate, and F1 score are determined using standard definitions.

Executing and evaluating Orchard and Sapling* on low-coverage DNA sequencing data

To evaluate Orchard and Sapling*, we simulated tumor phylogenies using an identical procedure as in Simulation details, but modified the simulation parameters and the tree moves in Sapling*. Specifically, we simulated 40 tumor phylogenies with $n \in \{50, 100, 250, 500\}$ mutations, $m \in \{50, 100\}$ samples, and $c = 20$ read coverage across $s \in \{1, \dots, 5\}$ random number generator seeds. To modify the tree moves in Sapling*, we considered the expanded set of moves used in Orchard (Kulman et al., 2024) and fastBE (Schmidt and Raphael, 2024), which upon the addition of a new mutation to the current tree, considered all possible (in worst-case 2^n) insertions that respected the current tree's partial order.

Down-sampling read counts on a mouse model of colorectal cancer

To down-sample the POP66 colorectal data to a lower 20× coverage, we took the original variant and total read count matrices $\mathbf{V}, \mathbf{D}$ and constructed the observed frequency matrix $\tilde{\mathbf{F}} = [\tilde{f}_{pj}]$ where $\tilde{f}_{pj} = v_{pj}/d_{pj}$. Then, we generated a synthetic m-by-n total read count matrix $\mathbf{D}' = [d'_{pj}]$ by drawing $d'_{pj} \sim \text{Poisson}(20)$. Using the total read count matrix, we then constructed the down-sampled variant matrix $\mathbf{V}' = [v'_{pj}]$ by drawing the variant reads using the observed frequencies: $v'_{pj} \sim \text{Binomial}(\tilde{f}_{pj}, d'_{pj})$. The result of this process was the original variant and total read count matrices $\mathbf{V}, \mathbf{D}$ along with the down-sampled variant and total read count matrices $\mathbf{V}', \mathbf{D}'$.

For the subsequent analysis, we built phylogenies by passing in the down-sampled read count matrices $\mathbf{V}'$ and $\mathbf{D}'$ to Orchard and Sapling*, obtaining tumor phylogenies $\mathcal{T}_1$ and $\mathcal{T}_2$ respectively. We then measured the concordance of both trees with the original and down-sampled data by computing $L^*(\mathcal{T}_i \mid \mathbf{V}, \mathbf{D})$ and $L^*(\mathcal{T}_i \mid \mathbf{V}', \mathbf{D}')$ for $i = 1, 2$ under both the binomial log-likelihood and ℓ_2 loss (Table 2).

fastppm implementation and data availability

fastppm is an open-source implementation of the TSDDP algorithms for the ℓ_2, binomial (via PLA, PPLA and ADDM) and beta-binomial (via PLA and PPLA) loss functions. *fastppm* is implemented in C++ as both a shared library and command-line interface. *fastppm* provides Python bindings using PyBind. *fastppm* is available at: `github.com/elkebir-group/fastppm.git`. Simulated and real data is available at `github.com/elkebir-group/fastppm-data.git`.

Supplementary Proofs

Proof of Eq. (5). The recurrence relation in (5) follows from the following chain of equalities:

$$\begin{aligned} J_i(\gamma) &= \max_{\boldsymbol{\alpha}\geq 0}\{\textstyle\sum_{j\in D(i)} h_j(\alpha_j - \alpha_{\pi(j)}) : \alpha_{\pi(i)} = \gamma\} \\ &= \max_{\alpha_i\geq 0}\{\max_{\boldsymbol{\alpha}_{-i}\geq 0}\{\textstyle\sum_{j\in D(i)} h_j(\alpha_j - \alpha_{\pi(j)})\} : \alpha_{\pi(i)} = \gamma\} \\ &= \max_{\alpha_i\geq 0}\{h_i(\alpha_i - \gamma) + \max_{\boldsymbol{\alpha}_{-i}\geq 0}\{\textstyle\sum_{j\in D(i)\setminus\{i\}} h_j(\alpha_j - \alpha_{\pi(j)})\}\} \\ &= \max_{\alpha_i\geq 0}\{h_i(\alpha_i - \gamma) + \textstyle\sum_{j\in\delta(i)} J_j(\alpha_i)\}. \end{aligned}$$

The first equality holds by definition of $J_i(\cdot)$, the second equality holds by defining $\boldsymbol{\alpha}_{-i} = (\alpha_0, \ldots, \alpha_{i-1}, \alpha_{i+1}, \ldots, \alpha_n)$, the third equality holds by constancy of $h_i(\cdot)$, and the final equality holds by the separability of the sum with α_i fixed. □

Proof of Observation 2. This follows from the stronger statement that $f(x) = \max_{y\in C}\{f_1(y - x) + f_2(y)\}$ is concave, provided that f_1 and f_2 are concave, and C is convex. To see this, observe that $f(x)$ is the suprememal projection of the concave function $f_1(y - x) + f_2(y)$ onto the y coordinate, over a convex set. It is well-known that supremal projections of concave functions (equivalently infimal projections for convex functions) preserve concavity. See equation (3.16) in Boyd (Boyd and Vandenberghe, 2004) for a reference and proof.

Using the preceding result, the observation follows from induction over $\mathcal{T}$. In the case where i is a leaf, $J_i(\gamma) = \max_{\alpha_i\geq 0}\{h_i(\alpha_i - \gamma)\}$ and the result holds by setting $f_1 = h_i$ and $f_2 = 0$. Since concavity is preserved under summation, inductively, we have that $J_i(\gamma) = \max_{\alpha_i\geq 0}\{h_i(\alpha_i - \gamma) + \sum_{j\in\delta(i)} J_j(\alpha_i)\}$ is concave upon setting $f_1 = h_i$ and $f_2 = \sum_{j\in\delta(i)} J_j$. □

Proof of Theorem 4. Let $\mathbf{f} = \arg\min_{\mathbf{f}}\{\tilde{L}(\mathbf{f}) : \mathbf{f} \text{ satisfies (SC)}\}$ and $\mathbf{f}' = \arg\min_{\mathbf{f}}\{L(\mathbf{f}) : \mathbf{f} \text{ satisfies (SC)}\}$. Let $\mathbf{f}''$ be the nearest point (under the $\|\cdot\|_1$ norm) to $\mathbf{f}$ on the n-dimensional grid $G = \{\frac{1}{k}, \frac{2}{k}, \ldots, \frac{k-1}{k}\}^n$. By construction, $|\mathbf{f}_i - \mathbf{f}''_i| \leq \frac{1}{2k}$. Consequently,

$$\begin{aligned} L(\mathbf{f}) - \tilde{L}(\mathbf{f}) &= L(\mathbf{f}) - \tilde{L}(\mathbf{f}'') + \tilde{L}(\mathbf{f}'') - \tilde{L}(\mathbf{f}) && (28)\\ &\leq |L(\mathbf{f}) - \tilde{L}(\mathbf{f}'') + \tilde{L}(\mathbf{f}'') - \tilde{L}(\mathbf{f})| && \text{by definition of } |\cdot| \quad (29)\\ &\leq |L(\mathbf{f}) - L(\mathbf{f}'')| + |L(\mathbf{f}'') - \tilde{L}(\mathbf{f})| && \text{by the triangle inequality,} \quad (30)\\ &= |\sum_{i=1}^n L_i(\mathbf{f}_i) - \sum_{i=1}^n L_i(\mathbf{f}''_i)| + |L(\mathbf{f}'') - \tilde{L}(\mathbf{f})| && \text{by definition of } L, \quad (31)\\ &\leq \frac{n\beta}{2k} + |L(\mathbf{f}'') - \tilde{L}(\mathbf{f})| && \text{by } \beta\text{-Lipschitz continuity of } L_i, \quad (32)\\ &= \frac{n\beta}{2k} + |L(\mathbf{f}'') - \tilde{L}(\mathbf{f}'') + \tilde{L}(\mathbf{f}'') - \tilde{L}(\mathbf{f})| && \text{by adding zero,} \quad (33)\\ &= \frac{n\beta}{2k} + |\tilde{L}(\mathbf{f}'') - \tilde{L}(\mathbf{f})| && \text{since } \mathbf{f}'' \text{ is on } G, \quad (34)\\ &\leq \frac{n\beta}{2k} + \frac{n\beta}{2k} && \text{by } \beta\text{-Lipschitz continuity of } \tilde{L}_i. \quad (35) \end{aligned}$$

Using both the fact that $L(\mathbf{f}') \geq \tilde{L}(\mathbf{f})$ and $L(\mathbf{f}') \leq L(\mathbf{f})$, we have

$$0 \leq L(\mathbf{f}') - \tilde{L}(\mathbf{f}) \leq L(\mathbf{f}) - \tilde{L}(\mathbf{f}) \leq \frac{n\beta}{k}. \quad (36)$$

Consequently, $0 \leq L^*(\mathcal{T}) - \tilde{L}^*(\mathcal{T}) \leq \frac{n\beta}{k}$, completing the proof. □

Supplementary Figures

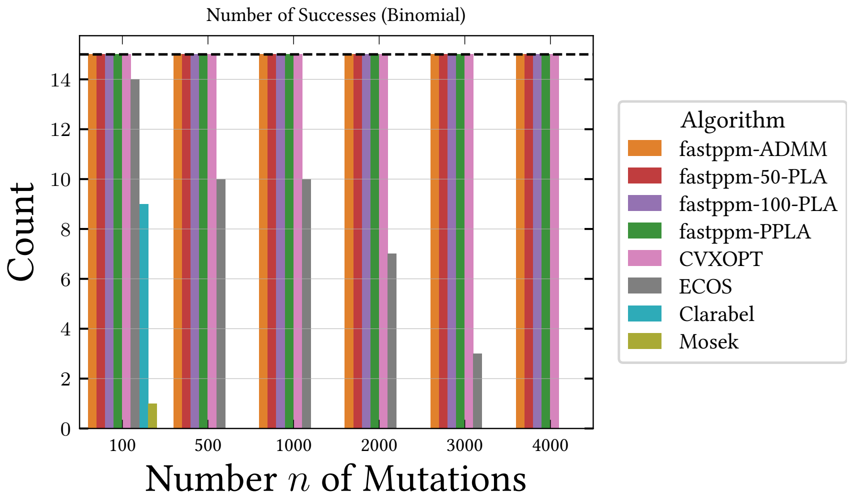

Fig. S1. Number of successes of existing algorithms for the Perfect Phylogeny Regression problem across 90 tumor phylogenies for the binomial negative log-likelihood loss function. For the ℓ_2 loss, all algorithms terminated successfully across all 90 instances and are excluded.

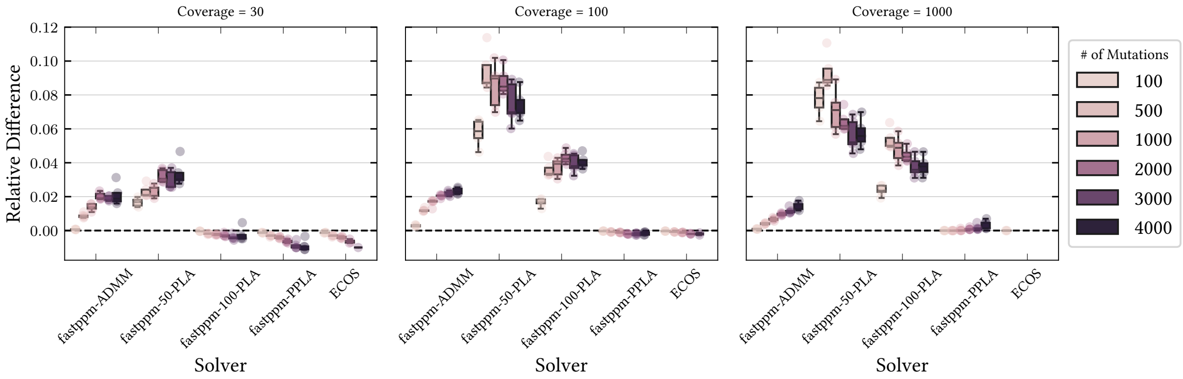

Fig. S2. Relative difference $\epsilon := \frac{L^* - L^*_{\text{CVXOPT}}}{L^*}$ in the binomial log-likelihood of the inferred frequencies between all solvers and CVXOPT. L^* denotes the binomial log-likelihood of the inferred frequencies for a particular solver and L^*_{CVXOPT} denotes the binomial log-likelihood of the inferred frequencies for CVXOPT.

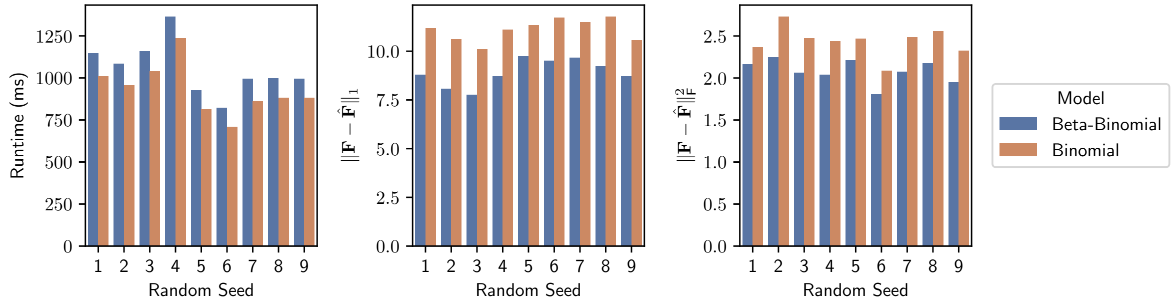

Fig. S3. Runtime (ms) of PPLA and difference in the inferred and true frequency matrices when inferring frequencies using a beta-binomial and binomial loss function. Simulated data has $n = 50$ mutations, $s = 100$ samples, and $c = 20$ coverage. Variant read counts were generated using the beta-binomial distribution with precision parameter $s = 2.0$.

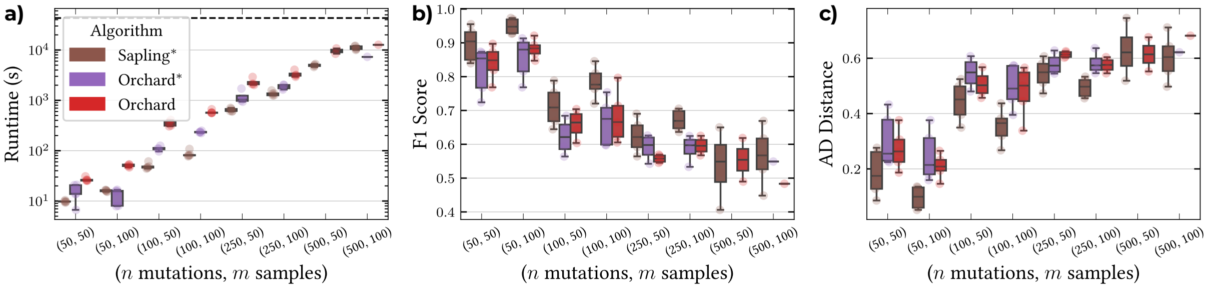

Fig. S4. **(Left-to-Right)** False positive rate, false negative rate, ancestor-descendant distance, and parent-child distance between true and inferred phylogenies of Sapling (Qi and El-Kebir, 2024) and Sapling* across 120 simulated tumor phylogenies. Sapling* is the result of replacing the CVXOPT solver for the binomial negative log-likelihood loss in Sapling with *fastppm*-ADMM.

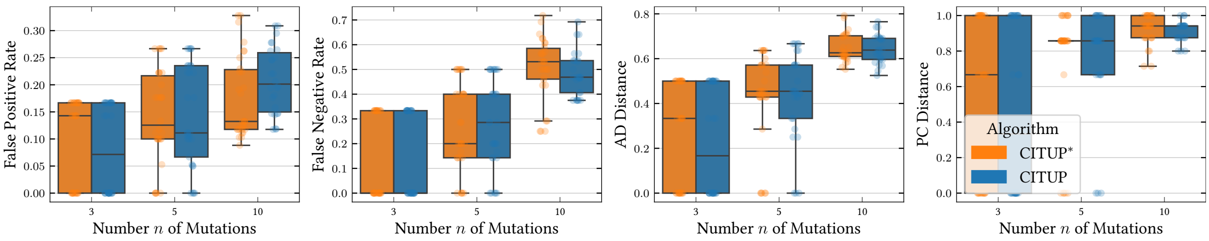

Fig. S5. **(Left-to-Right)** False positive rate, false negative rate, ancestor-descendant distance, and parent-child distance between true and inferred phylogenies of CITUP (Malikic et al., 2015) and CITUP* across 180 simulated tumor phylogenies. CITUP* is the result of replacing the CPLEX solver for the ℓ_2 loss in CITUP with *fastppm*-L2.

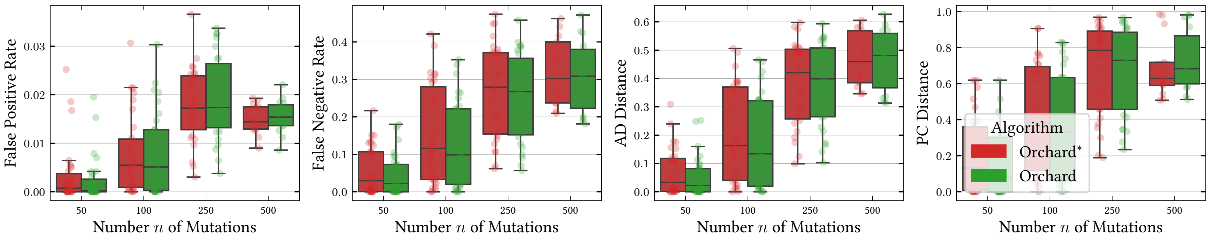

Fig. S6. **(Left-to-Right)** False positive rate, false negative rate, ancestor-descendant distance, and parent-child distance between true and inferred phylogenies of Orchard (Kulman et al., 2024) and Orchard* across 120 simulated tumor phylogenies. Orchard* is the result of replacing the projectppm solver for the ℓ_2 loss in Orchard with *fastppm*-L2.

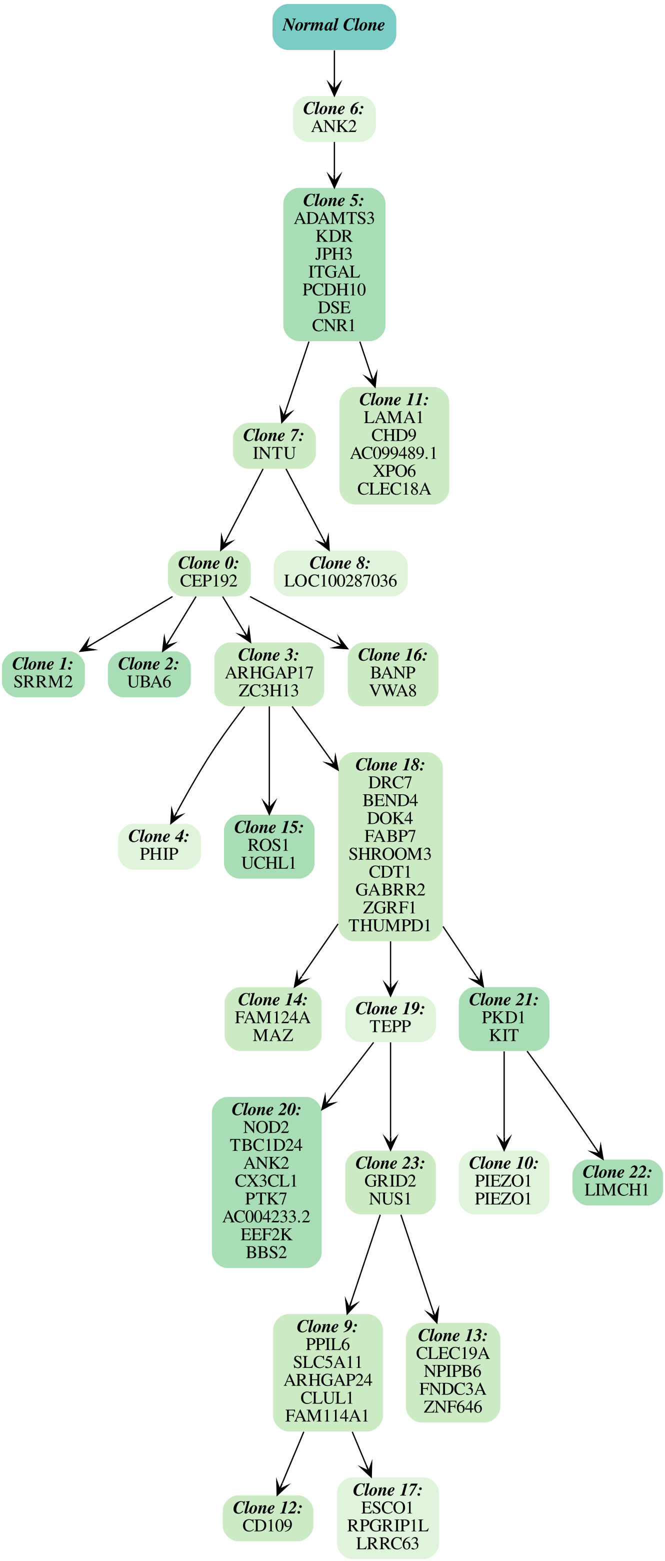

Fig. S7. The phylogeny inferred by Orchard (Kulman et al., 2024) on patient-derived xenograft POP66 (Rehman et al., 2021). For simplicity of visualization, degree-2 nodes are collapsed into clusters.

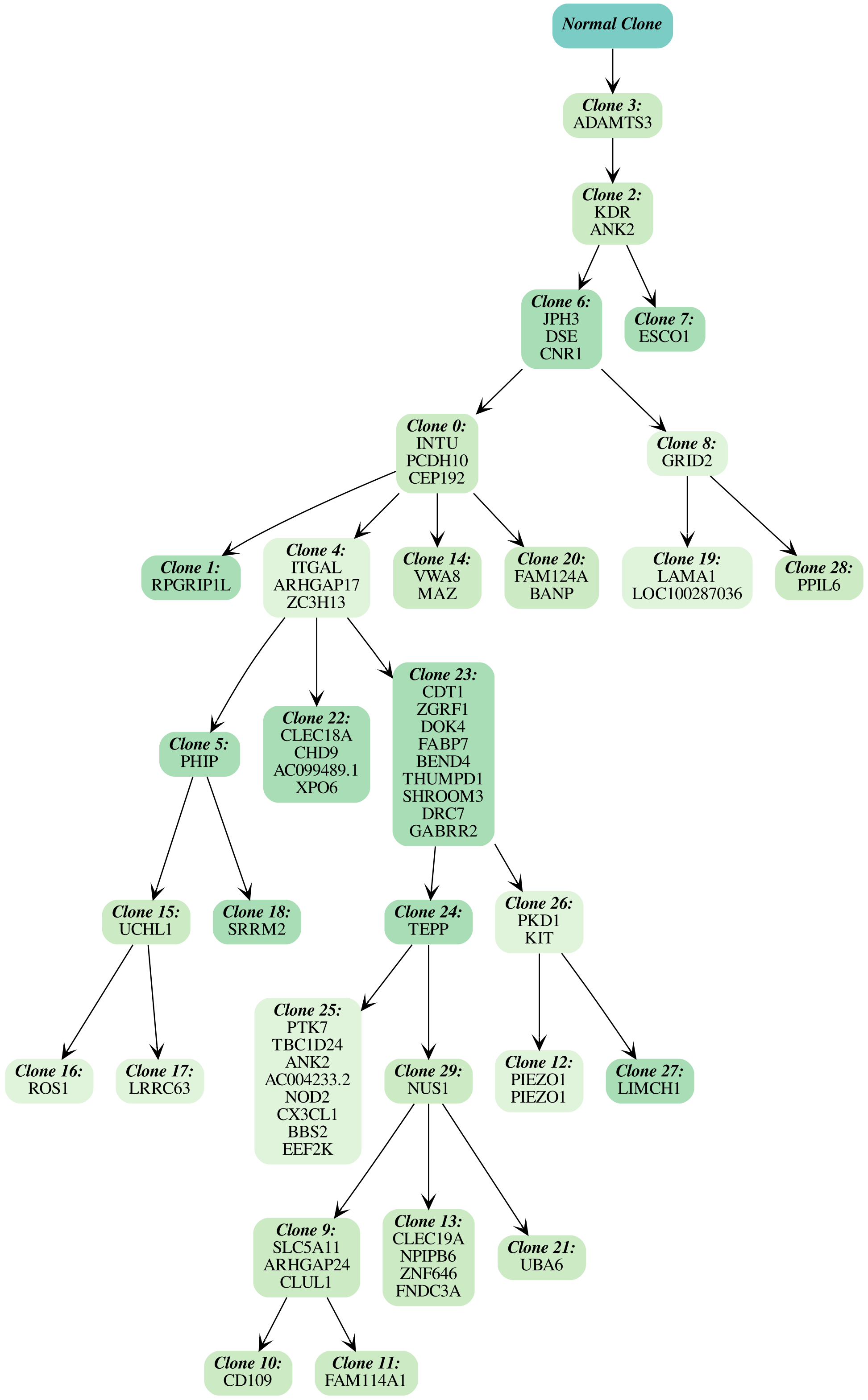

Fig. S8. The phylogeny inferred by Orchard* on patient-derived xenograft POP66 (Rehman et al., 2021). For simplicity of visualization, degree-2 nodes are collapsed into clusters.

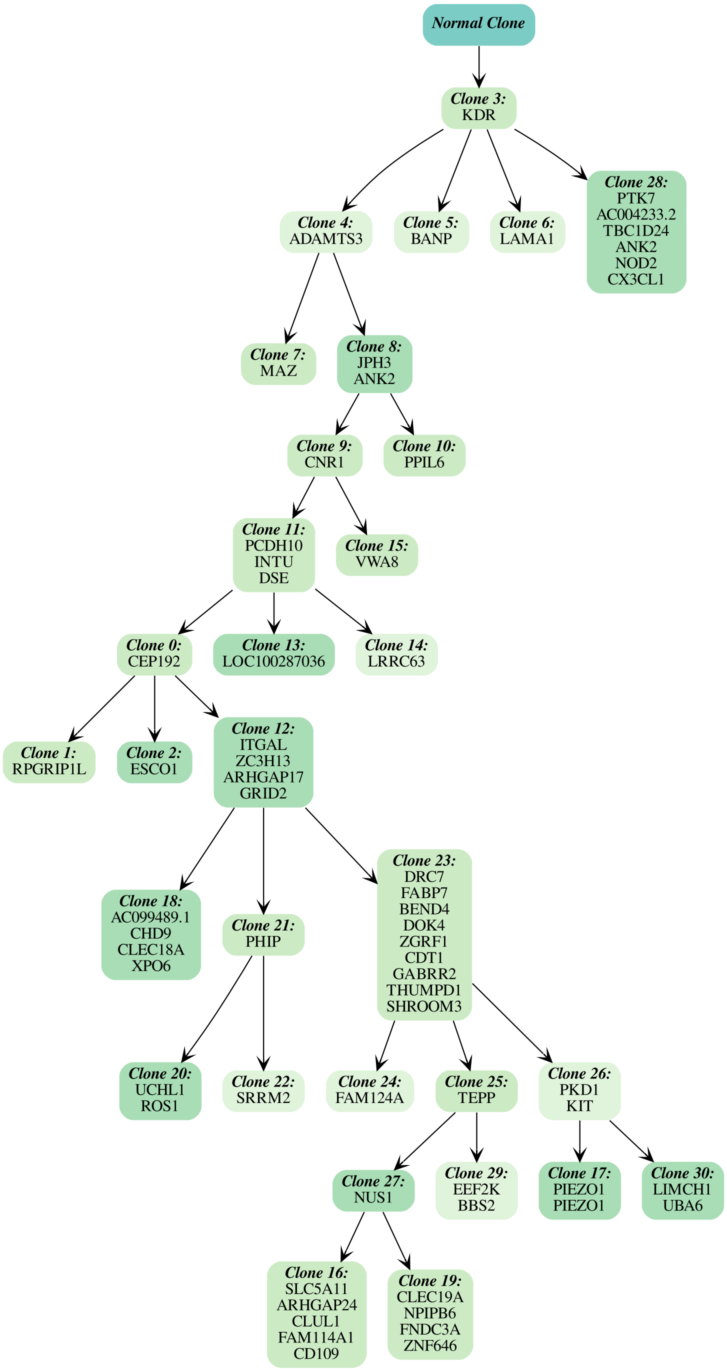

Fig. S9. The phylogeny inferred by Sapling* on patient-derived xenograft POP66 (Rehman et al., 2021). For simplicity of visualization, degree-2 nodes are collapsed into clusters.
